# DncV Synthesizes Cyclic GMP-AMP and Regulates Biofilm Formation and Motility in *Escherichia coli* ECOR31

**DOI:** 10.1128/mBio.02492-18

**Published:** 2019-03-05

**Authors:** Fengyang Li, Annika Cimdins, Manfred Rohde, Lothar Jänsch, Volkhard Kaever, Manfred Nimtz, Ute Römling

**Affiliations:** a Department of Microbiology, Tumor and Cell Biology, Karolinska Institutet, Stockholm, Sweden; b Central Facility for Microscopy, Helmholtz Centre for Infection Research, Braunschweig, Germany; c Cellular Proteomics, Helmholtz Centre for Infection Research, Braunschweig, Germany; d Research Service Centre Metabolomics, Hannover Medical School, Hannover, Germany; Biozentrum/University of Basel; University of Würzburg

**Keywords:** CsgD, DncV, biofilm formation, cGAMP, cyclic di-GMP, motility, rdar

## Abstract

Cyclic dinucleotides (cDNs) act as intracellular second messengers, modulating bacterial physiology to regulate the fundamental life style transition between motility and sessility commonly known as biofilm formation. Cyclic GMP-AMP (cGAMP), synthesized by the dinucleotide cyclase DncV, is a newly discovered cDN second messenger involved in virulence and chemotaxis in Vibrio cholerae O1 biovar El Tor. Here we report a novel role for horizontally transferred DncV in cGAMP production and regulation of biofilm formation and motility in the animal commensal strain Escherichia coli ECOR31. ECOR31 expresses a semiconstitutive temperature-independent rdar (red, dry, and rough) morphotype on Congo red agar plates characterized by the extracellular matrix components cellulose and curli fimbriae which requires activation by the major biofilm regulator CsgD and cyclic di-GMP signaling. In contrast, C-terminal His-tagged DncV negatively regulates the rdar biofilm morphotype and cell aggregation via downregulation of *csgD* mRNA steady-state level. Furthermore, DncV sequentially promotes and inhibits adhesion to the abiotic surface after 24 h and 48 h of growth, respectively. DncV also suppresses swimming and swarming motility posttranscriptional of the class 1 flagellum regulon gene *flhD*. Purified DncV produced different cDNs, cyclic di-GMP, cyclic di-AMP, an unknown product(s), and the dominant species 3′3′-cGAMP. *In vivo*, only the 3′3′-cGAMP concentration was elevated upon short-term overexpression of *dncV*, making this work a first report on cGAMP production in E. coli. Regulation of rdar biofilm formation and motility upon overexpression of untagged DncV in combination with three adjacent cotransferred gene products suggests a novel temperature-dependent cGAMP signaling module in E. coli ECOR31.

## Abstract (precis)

The ability of bacteria to sense and respond to environmental signals is critical for survival. Bacteria use cyclic dinucleotides as second messengers to regulate a number of physiological processes, such as the fundamental life style transition between motility and sessility (biofilm formation). cGAMP, which is synthesized by a dinucleotide cyclase called DncV, is a newly discovered second messenger involved in virulence and chemotaxis in the Vibrio cholerae biovar El Tor causing the current 7th cholera pandemic. However, to what extent cGAMP exists and participates in physiological processes in other bacteria is still unknown. In this study, we found an elevated cGAMP level to possibly regulate biofilm formation and motility in the animal commensal E. coli strain ECOR31. Thus, we detected a novel role for cGAMP signaling in regulation of physiological processes other than those previously reported in proteobacterial species.

## INTRODUCTION

**C**yclic dinucleotides (cDNs), two nucleotides covalently linked via two phosphodiester bonds, are ubiquitous second messengers in bacteria and metazoa (1, 2). Four cDNs have been discovered so far: cyclic di-GMP (c-di-GMP), cyclic di-AMP (c-di-AMP), and, recently, two hybrid cyclic GMP-AMPs (cGAMP). Cyclic GAMP exists as a canonical and a noncanonical isomer, with 3′3′-cGAMP mainly described in bacteria, while 2′3′-cGAMP is an innate immune signaling molecule in eukaryotes (3–5).

The different bacterial cDNs have distinct physiological functions. Cyclic di-GMP is an ubiquitous bacterial second messenger involved in regulation of the transition between complex life style changes on the single-cell level, such as the transition between sessility (biofilm formation) and motility, which translates to the transition between acute and chronic virulence in host interaction (4, 6–8). On the other hand, c-di-AMP is involved in the maintenance of osmohomeostasis and abiotic stress, monitoring of DNA integrity, cell wall synthesis, and virulence (9–12). Cyclic GAMP was only recently identified in Vibrio cholerae O1 biovar El Tor causing the 7th pandemic of cholera as a second messenger molecule, which promotes infectivity through repression of chemotaxis and enhanced intestinal colonization (3). Cyclic GAMP is mainly synthesized by the DncV dinucleotide cyclase, which is sequentially and structurally distinct from major c-di-GMP and c-di-AMP cyclases. In another physiological process, cGAMP signaling regulates genes associated with extracellular electron transfer in the deltaproteobacterial *Geobacter* genus (13, 14).

In eukaryotes, noncanonical 2′3′-cGAMP is synthesized by the cyclic GMP-AMP synthase (cGAS) in response to cytoplasmic sensing of DNA (15–17). The cGAS product 2′3′-cGAMP subsequently stimulates the innate immune adaptor STING and thereby promotes an innate immune response and senescence (15, 17, 18). In contrast to mammalian cGAS, bacterial DncV is believed to produce 3′3′-cGAMP exclusively (3, 19, 20). Both bacterial cGAMP and eukaryotic cGAMP, as also seen with c-di-GMP and c-di-AMP, bind not only to STING but also to various other metazoan receptors to promote a broad physiological response (2, 21). Besides the dominant product cGAMP, V. cholerae-derived DncV generates c-di-GMP and c-di-AMP as side products, suggesting that one enzyme can synthesize multiple cDNs *in vivo* to affect bacterial physiology (3).

The ubiquitous c-di-GMP is a major stimulator of biofilm formation and a repressor of flagellum biosynthesis and function in Gram-negative and Gram-positive bacteria (6, 22, 23). In agar plate-grown *Enterobacteriaceae*, c-di-GMP activates a distinct multicellular biofilm behavior termed the rdar (red, dry, and rough) morphotype (24, 25). The major target of c-di-GMP signaling in Salmonella enterica serovar Typhimurium and Escherichia coli is therefore the orphan transcriptional regulator CsgD, which subsequently activates expression of the extracellular biofilm matrix components amyloid curli fimbriae and, indirectly, via the diguanylate cyclase AdrA, the exopolysaccharide cellulose (26–28). On the other hand, flagellum-mediated swimming and swarming motility is repressed by c-di-GMP (6).

Bioinformatic analyses showed that homologs of DncV are present in bacteria other than V. cholerae biovar El Tor (3), including animal-commensal E. coli strain ECOR31 from the ECOR reference collection (29). In ECOR31, DncV_ECOR31_ is located on a 35-kb horizontally transferred composite genomic island (designated RB-HPI_ECOR31_ [RB, right border; HPI, high-pathogenicity island]) at the right border of a widely distributed HPI present in uropathogenic strain E. coli CFT073, *Yersinia* species, and other *Enterobacteriaceae* that cause extraintestinal infections (30).

In this work, we report that cDN cyclase DncV_ECOR31_ from E. coli ECOR31 predominantly synthesizes the novel second messenger 3′3′-cGAMP and, in combination with three adjacent horizontally cotransferred gene products, downregulates rdar biofilm formation and flagellum-mediated motility in a temperature-dependent manner. This study thus reveals a novel potential role for a recently horizontally transferred cGAMP signaling module in regulation of physiological processes other than those previously reported in proteobacterial species.

## RESULTS

### ECOR31 displays a *csgD*-dependent rdar morphotype and *csgD-*independent motility.

**FIG 1:**
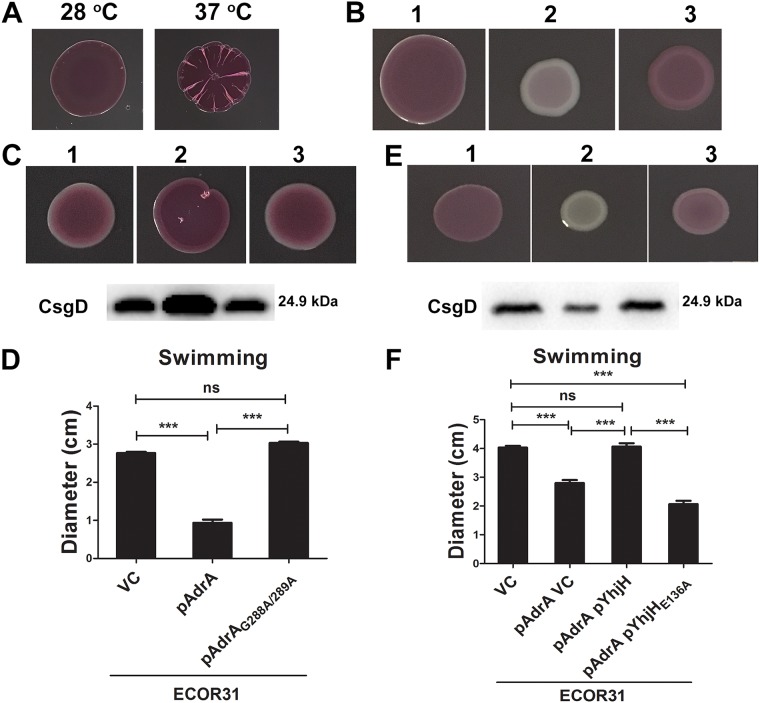
The sessility/motility life style change of E. coli ECOR31 is regulated by *csgD* and the c-di-GMP signaling network. (A) Rdar morphotype of E. coli ECOR31 on Congo red agar plates after 24 h of incubation at 28°C and 37°C. (B) Rdar morphotype of E. coli ECOR31 upon deletion and overexpression of *csgD*. 1, ECOR31 VC; 2, ECOR31 Δ*csgD* VC; 3, ECOR31 Δ*csgD* pCsgD (VC, vector control pBAD28; pCsgD, *csgD* cloned in pBAD28). (C and D) rdar morphotype and CsgD production (C) and swimming motility (D) of E. coli ECOR31 upon overexpression of the diguanylate cyclase AdrA and its catalytic mutant AdrA_G288A/G289A_. 1, ECOR31 VC; 2, ECOR31 pAdrA; 3, ECOR31 pAdrA_G288A/G289A_ (VC, pBAD28; pAdrA, AdrA cloned in pBAD28). (E) Effect of overexpression of the c-di-GMP-specific phosphodiesterase YhjH in E. coli ECOR31 on rdar morphotype and CsgD production. 1, ECOR31 VC; 2, ECOR31 pYhjH; 3, ECOR31 pYhjH_E136A_ (VC, pSRKGm). B, C, E: Only colony morphotypes from the same plate are compared. (F) Swimming motility upon coexpression of the diguanylate cyclase AdrA and the c-di-GMP phosphodiesterase YhjH. ECOR31 VC, pBAD28 and pSRKGm; pAdrA VC, pAdrA and pSRKGm (pAdrA, AdrA cloned in pBAD28; pYhjH, YhjH cloned in pSRKGm). Bars in D and F represent the mean values, with vertical error bars representing standard deviations (SD). Differences between mean values were assessed by an unpaired two-tailed Student's *t* test (ns, not significant; ***, *P < *0.001 [compared to ECOR31 vector control]).

Strain E. coli ECOR31 (ATCC 35350) was isolated from the feces of a leopard (29). The rdar biofilm morphotype is commonly expressed by *S.* Typhimurium and E. coli strains (see Fig. S1A in the supplemental material) (24, 31). We first investigated whether ECOR31 produces the rdar morphotype. Indeed, ECOR31 colonies displayed a semiconstitutive temperature-independent rdar biofilm morphotype on Congo red agar plates at 28°C and 37°C (Fig. 1A). Deletion of *csgBA* genes encoding the major and minor subunits CsgA and CsgB, respectively, of amyloid curli fimbriae and *bcsA* coding for the catalytic subunit of the cellulose synthase resulted in the expected color change of the agar-grown colony, thus demonstrating that rdar colonies of ECOR31 express the extracellular matrix components curli and cellulose at both temperatures (Fig. S1B) (32).

10.1128/mBio.02492-18.1 FIG S1 Characterization of major regulatory pathways and rdar morphotype components in E. coli ECOR31. (A) Conventional regulatory network of the rdar colony morphology biofilm. The biofilm transcriptional regulator CsgD activates curli fimbriae, and cellulose biosynthesis through the c-di-GMP synthesizing di-guanylate cyclase AdrA. The second messenger cyclic di-GMP activates biofilm formation further through *csgD* expression and repression of flagellar-mediated motility by binding to the PilZ domain of the c-di-GMP receptor YcgR and to the ATPase FliI. The stress sigma factor RpoS and the response regulator OmpR are other major activators of *csgD* expression. (B) Amyloid curli fimbriae and cellulose are extracellular matrix components of the rdar morphotype of E. coli ECOR31 as assessed by mutant analysis of genes coding for the major curli subunit CsgA and the cellulose synthase BcsA. (C) The response regulator OmpR and the stationary-phase sigma factor RpoS are partially required for rdar morphotype expression. The rdar (red, dry, and rough) morphotype is characterized by expression of extracellular matrix components curli fimbriae and cellulose. The ras (red and smooth) morphotype displays diminished expression. pdar, pink, dry, and rough morphotype (cellulose^+^). The pas (pink and smooth) morphotype (cellulose^+^) displays diminished expression. bdar, brown, dry and rough morphotype (curli^+^); saw, smooth and white morphotype (cellulose^−^, curli^−^); gas (gray and smooth) morphotype, uncharacterized. A 5-µl volume of cell suspension was spotted onto a salt-free LB plate containing Congo red and incubated at 28°C and 37°C for 24 h. Only colony morphotypes from the same plate were compared. Download FIG S1, TIF file, 3.0 MB. Copyright © 2019 Li et al. 2019 Li et al. This content is distributed under the terms of the Creative Commons Attribution 4.0 International license.

Regulation of the rdar morphotype is positively controlled by the transcriptional regulator CsgD (24, 25). A *csgD* deletion mutant of ECOR31 displayed a saw (smooth and white) colony morphology at both temperatures, indicating lack of curli and cellulose production. The rdar morphotype, though, was restored upon overexpression of *csgD* from the pBAD28 vector (Fig. 1B), thus substantiating the prediction of *csgD*-dependent rdar morphotype expression of ECOR31, in agreement with previous findings for most E. coli strains (32, 33).

We subsequently investigated whether additional basic regulatory principles of rdar morphotype expression apply to ECOR31 (Fig. S1A). Major global regulators of the rdar morphotype that affect *csgD* transcription are the response regulator OmpR and the stationary-phase sigma factor RpoS (25, 34). Analysis of the *ompR* and *rpoS* deletion mutants showed expression of the temperature-independent rdar morphotype of ECOR31 to partially require OmpR and RpoS (Fig. S1C).

In E. coli K-12, *csgD* targets flagellum regulon genes and inhibits swimming motility (35, 36). The swimming motility of ECOR31, though, was not altered upon either overexpression or deletion of *csgD* compared to the ECOR31 wild-type (Fig. S2A), suggesting swimming motility of ECOR31 to be *csgD* independent under the assay conditions used in this work (37).

10.1128/mBio.02492-18.2 FIG S2 Swimming motility, rdar biofilm formation, and CsgD production of E. coli ECOR31 upon overexpression of diguanylate cyclases (DGCs) and c-di-GMP-specific phosphodiesterases (PDEs). (A) Swimming motility of ECOR31 and its *csgD* deletion mutant. VC, pBAD28. (B) Rdar morphotype, CsgD expression, and swimming motility of ECOR31 upon overexpression of the DGC YdeH. VC, pBAD28. (C) Rdar morphotype and CsgD expression of ECOR31 upon overexpression of the PDE YE2225. VC, pSRKGm. (D) Swimming motility of ECOR31 upon overexpression of the PDEs YhjH and YE2225. VC, pSRKGm. (E) Swimming motility of ECOR31 upon overexpression of the DGC YdeH altered by coexpression of the PDEs YhjH and YE2225. ECOR31 VC, pBAD28 and pSRKGm; pYdeH VC, pYdeH and pSRKGm. pYdeH, YdeH cloned in pBAD28; pYhjH, YhjH cloned in pSRKGm; pYE2225, = YE2225 cloned in pSRKGm. Overnight cultures grown on LB agar plates were resuspended to the same cell density (OD_600_ = 5). For the swimming assay, 3 µl were inoculated into soft agar plates containing 1% tryptone, 0.5% NaCl and 0.25% agar and the swimming diameter was measured after 7 h at 28°C. For rdar morphotype assessment, 5 µl were spotted onto LB without salt plate containing Congo red and incubated at 28°C for 24 h. Mean values represent results from three experiments performed with three technical replicates, and error bars represent SD. ns, not significant; *, *P < *0.05; ***, *P < *0.001 (compared to ECOR31 VC; unpaired two-tailed Student’s *t* test). Download FIG S2, TIF file, 2.9 MB. Copyright © 2019 Li et al. 2019 Li et al. This content is distributed under the terms of the Creative Commons Attribution 4.0 International license.

As a major target, expression of the CsgD transcriptional regulator is positively regulated by the second messenger c-di-GMP, while flagellum-mediated swimming and swarming motility is repressed by the c-di-GMP network (6). To assess whether this common principle of rdar biofilm versus motility life style regulation by the c-di-GMP regulatory network holds also for the ECOR31 strain, two well-established diguanylate cyclases (DGCs), AdrA (6) and YdeH (38), and two c-di-GMP-dependent phosphodiesterases (PDEs), YhjH and YE2225 (39), were overexpressed. In agreement with previous findings, overexpression of the diguanylate cyclases AdrA and YdeH upregulated the rdar morphotype and CsgD expression and downregulated swimming motility, while their catalytic mutants had no effect (Fig. 1C and D; see also Fig. S2B). In contrast, overexpression of c-di-GMP phosphodiesterases YhjH and YE2225 downregulated the rdar morphotype and CsgD expression, whereas, their catalytic mutants again had no effect (Fig. 1E; see also Fig. S2C). These results showed that c-di-GMP signaling activates the rdar morphotype as demonstrated previously for other E. coli and *S.* Typhimurium strains (6, 40).

Overexpression of motility-specific YhjH and YE2225 phosphodiesterases had no effect on swimming motility at 37°C and 28°C, though (Fig. S2D and data not shown). We had previously observed that swimming motility in *S.* Typhimurium was also barely affected upon overexpression of these PDEs under similar experimental conditions (6, 39). We assumed that the c-di-GMP dedicated to swimming motility was entirely depleted, which prevented evaluation of an effect of c-di-GMP hydrolysis even by the motility-dedicated PDE YhjH. To assess in an alternative way whether c-di-GMP degradation relieves the inhibitory effect of c-di-GMP on flagellum-mediated swimming motility, we inhibited motility by overexpression of a DGC (Fig. 1D; see also Fig. S2B) and subsequently coexpressed a PDE to relieve suppression. Coexpression of the diguanylate cyclase AdrA with increasing amounts of the PDE YhjH in ECOR31 gradually relieved the inhibitory effect of the DGC AdrA on motility (Fig. 1F and data not shown). Expression of the catalytic mutant YhjH_E136A_, though, maintained repression, demonstrating that the catalytic activity of the YhjH PDE is required to stimulate swimming motility (Fig. 1F). We observed similar results when we coexpressed the DGC YdeH together with PDE YhjH or YE2225, including their catalytic mutants (Fig. S2E). Thus, we conclude that c-di-GMP inhibits flagellum-mediated swimming motility in E. coli ECOR31 as observed previously in other E. coli strains and bacterial species (6, 22).

### *dncV* downregulates rdar morphotype and *csgD* expression.

In E. coli ECOR31, the 35-kb RB-HPI genomic island has been inserted at the right border of the HPI (30). Bioinformatic analyses indicated that the right border of the RB-HPI island encodes multiple nucleotide signaling components (data not shown) (41). DncV_ECOR31_, the homolog of V. cholerae dinucleotide cyclase DncV_Vcholerae_, is flanked upstream and downstream by genes horizontally cotransferred from the V. cholerae VSP-1 island (3). However, as in V. cholerae, DncV homologs are infrequently encoded by E. coli strains, with DncV homologs with >80% identity found in fewer than 30 sequenced E. coli strains, while >360 V. cholerae strains were found to harbor identical DncV proteins (BLAST search 26 November 2017, NCBI database). Results of phylogenetic analysis of representative DncV homologs supported classification in different subgroups (Fig. S3A). The occurrence of distinct homologs in isolates of the same species suggests horizontal transfer to have occurred more than once (Fig. S3A).

10.1128/mBio.02492-18.3 FIG S3 Phylogenetic and bioinformatic analysis of DncV homologs. (A) Maximum likelihood phylogenetic reconstruction of DncV homologs. DncV from E. coli ECOR31 was used as the query in the NCBI Blast search, and representative proteins (one representative per genus of equal homology) homologous over the entire length of the protein were retrieved. This search strategy collected proteins with sequence identity of >40%. Three DncV subgroups were identified. All proteins, with the exception of WP 063856357.1, contained the G[G/S]x_9-13_Dx[D/E] signature motif. (B) Sequence logo of group DncV1/2/3 homologs. (C) Sequence comparison of DncV from E. coli ECOR31 and V. cholerae biovar El Tor. Sequence alignment of DncV from E. coli ECOR31 (Ec_DncV) and V. cholerae (Vc_DncV) shows that DncV_ECOR31_ displays 61% identity and 74% similarity to DncV_Vcholerae_. In red, residues in DncV_ECOR31_ mutated to alanine (Q110A and D129A/D131A). Q_110_ binds to 2′OH of guanosine. D129, D131, and D194 (in green) coordinate the two divalent Mg^2+^ cations. G_111_S_112_ of the signature motif is indicated in blue. Download FIG S3, TIF file, 3.0 MB. Copyright © 2019 Li et al. 2019 Li et al. This content is distributed under the terms of the Creative Commons Attribution 4.0 International license.

**FIG 2:**
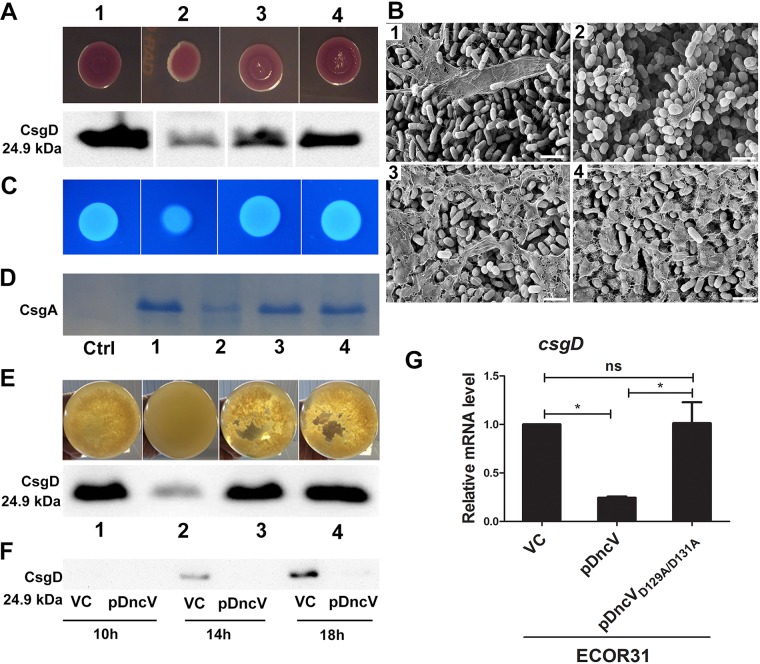
*dncV* downregulates the rdar morphotype, extracellular matrix components curli fimbriae and cellulose, cell aggregation, and *csgD* expression in E. coli ECOR31. (A) rdar morphotype and *csgD* expression in wild-type ECOR31 and upon overexpression of DncV and its catalytic mutants DncV_Q110A_ and DncV_D129A/D131A_. Cells were grown on a salt-free LB agar plate for 24 h at 28°C. Only colony morphotypes from the same plate and signals from the same Western blot are compared. (B) Scanning electron microscopy of cells from plate-grown colonies. Size bar, 2 µm. Cells were processed from a salt-free LB agar plate after 24 h of growth at 28°C. (C) Calcofluor white binding indicative for cellulose production in wild-type ECOR31 and upon overexpression of DncV and its catalytic mutants DncV_Q110A_ and DncV_D129A/D131A_ as visualized on calcofluor white containing salt-free LB agar plate after 24 h of growth at 28°C. (D) CsgA expression in wild-type ECOR31 and upon overexpression of DncV and its catalytic mutants DncV_Q110A_ and DncV_D129A/D131A_. Cells were grown on a salt-free LB agar plate at 28°C for 48 h. Semipurified cell extracts were treated with formic acid to depolymerize curli fimbriae into monomeric CsgA. Ctrl, negative-control ECOR31 VC, without formic acid treatment. (E) Cell aggregation and *csgD* expression in wild-type ECOR31 and upon overexpression of DncV and its catalytic mutants DncV_Q110A_ and DncV_D129A/D131A_ in liquid salt-free LB medium under microaerophilic conditions at 28°C after 16 h. Samples in panels A to E: 1, ECOR31 VC; 2, ECOR31 pDncV; 3, ECOR31 pDncV_Q110A_; 4, ECOR31 pDncV_D129A/D131A_ (VC, vector control pBAD28; pDncV, pDncV_Q110A_ and pDncV_D129A/D131A_ cloned in pBAD28). (F) CsgD is expressed late in the growth phase in wild-type ECOR31 and repressed upon overexpression of DncV. Cells were grown in liquid salt-free LB medium under microaerophilic conditions at 28°C, and samples were harvested after 10, 14, and 18 h of growth. VC, pBAD28; pDncV, DncV cloned in pBAD28. (G) Relative steady-state levels of *csgD* mRNA as estimated by qRT-PCR analysis. RNA was isolated from each group after 14 h. VC, pBAD28; pDncV, pDncV_D129A/D131A_, DncV, and variant cloned in pBAD28. Bars represent mean values, with error bars representing SD. Differences between mean values were assessed by two-tailed Student's *t* test (ns, not significant; *, *P < *0.05; **, *P < *0.01; ***, *P < *0.001 [compared to ECOR31 vector control]).

DncV_ECOR31_ shows 61% amino acid identity and 74% similarity to DncV_Vcholerae_ (NCBI BLAST; https://blast.ncbi.nlm.nih.gov/Blast.cgi?PROGRAM=blastp&PAGE) (Fig. S3C). To evaluate whether *dncV* affects expression of the rdar morphotype, we constructed a *dncV* deletion mutant. Deletion of *dncV*, though, showed no obvious effect on rdar morphotype expression (Fig. S4A). On the other hand, overexpression of C-terminal His-tagged DncV from the pBAD28 vector resulted in a light red and smooth colony on Congo red agar plates at 28°C and 37°C, clearly demonstrating suppression of the rdar morphotype compared with the ECOR31 wild-type vector control (Fig. 2A; see also Fig. S4B). In agreement with the colony morphotype, scanning and transmission electron microscopy (TEM) of agar-grown colonies that overexpressed DncV confirmed the diminished production of the extracellular matrix and exhibited cells with altered morphology and surface compared to the ECOR31 wild-type (Fig. 2B; see also Fig. S4B). In line with downregulation of the rdar morphotype, diminished production of CsgD, the major hub for activation of the rdar morphotype (Fig. 2A), and of *csgD*-regulated extracellular matrix components cellulose (as assessed by calcofluor white staining) and curli (as assessed by formic acid-dependent depolymerization of curli into monomers of the major curli subunit CsgA) was demonstrated (Fig. 2C and D).

10.1128/mBio.02492-18.4 FIG S4 Colony morphology and cell aggregation phenotypes of E. coli ECOR31 and derivatives. (A) Temperature-dependent effect of *dncV* and adjacent gene products on rdar morphotype expression. Data represent overexpression of *dncV* and differential complementation of an E. coli ECOR31 *dncV*::*Gm* mutant by adjacent gene products. Cells were grown on salt-free LB agar plates at 28°C for 24 h or at 37°C for 24 h. p78901, p*capVdncVvc0180vc0181* cloned in pBAD28; p*capVdncV*, *capV dncV* cloned in pBAD28. (B) Colony morphology and transmission electron microscopy of colony morphology of E. coli ECOR31 and derivatives. Colony morphologies of wild-type ECOR31 and upon overexpression of *dncV* and of its catalytic mutants DncV_Q109A_ and DncV_D129A/D131A_ grown on salt-free LB medium for 24 h at 37°C. ECOR31 wild-type cells and cells overexpressing the catalytic mutants DncV_Q109A_ and DncV_D129A/D131A_ are shown surrounded by a dense extracellular matrix throughout the colony, while overexpressing wild-type DncV led to formation of a colony which showed extracellular matrix-positive (colony interior) and extracellular matrix-negative (colony rim) areas. Δ*csgD*, extracellular matrix deficient negative control. (C) Cell aggregation and growth in liquid culture medium. Extensive cell aggregation dissolved upon overexpression of DncV. Cell aggregates started to be observed after 14 h of culturing. DncV was expressed from the pBAD28 vector by the use of 0.1% l-arabinose in the liquid salt-free LB medium at 28°C under microaerophilic conditions. VC, pBAD28; pDncV, DncV cloned in pBAD28. (D) Growth curve of ECOR31 upon overexpression of DncV and its catalytic mutant DncV_D129A/D131A_ in LB medium at 28°C and 37°C. Absorbance of the cell suspension was measured at OD_600_. Download FIG S4, TIF file, 3.0 MB. Copyright © 2019 Li et al. 2019 Li et al. This content is distributed under the terms of the Creative Commons Attribution 4.0 International license.

The crystal structure of DncV_Vcholerae_ demonstrated amino acid Q112 to be required for GTP binding, while amino acids D131 and D133 are critical catalytic residues that bind Mg^2+^ (20, 42, 43). To assess the effect of corresponding residues on DncV_ECOR31_ functionality, the amino acids Q110, D129, and D131 were replaced by alanine in DncV_ECOR31_. Expression of the DncV_D129A/D131A_ double mutant in the ECOR31 background did not alter rdar morphotype and CsgD production compared with the ECOR31 wild-type, indicating DncV_D129A/D131A_ to be nonfunctional. The DncV_Q110A_ mutant showed a rdar morphotype and CsgD production was less pronounced than in the ECOR31 wild-type, indicating partial functionality of DncV_Q110A_. Cellulose and curli production were not altered within the experimental error upon overexpression of DncV_D129A/D131A_ and DncV_Q110A_ compared with wild-type ECOR31 (Fig. 2A to D).

We assessed alternative modes of multicellular behavior and found that ECOR31 cells were arranged in pronounced aggregates in salt-free LB medium under aerobic and microaerophilic conditions (Fig. 2E and data not shown). We investigated the effect of *dncV* overexpression on cell aggregation and *csgD* expression in this alternative biofilm assay. Under microaerophilic conditions, visible cell aggregation started after approximately 14 h of growth, concomitant with CsgD production, and was fully developed after 16 to 18 h of growth (Fig. 2E and F; see also Fig. S4C). These findings are roughly consistent with observations in *S.* Typhimurium, where *csgD* is expressed in stationary-phase growth (44, 45). Overexpression of *dncV* led to resolution of aggregate formation and suppression of CsgD production as shown by Western blot analysis, in agreement with its effect on rdar morphotype expression (Fig. 2E and F; see also Fig. S4C). Upon overproduction of the two DncV mutants DncV_Q110A_ and DncV_D129A/D131A_, though, ECOR31 behaved in a manner similar to that seen with the wild-type strain (Fig. 2E).

Subsequently, we investigated on which level DncV, presumably through cGAMP, exerts its effect. Assessment of the *csgD* mRNA levels by reverse transcription-quantitative PCR (qRT-PCR) showed that the steady-state level of the mRNA was significantly reduced upon *dncV* overexpression but not upon overproduction of mutant protein DncV_D129A/D131A_ (Fig. 2G). In conclusion, these data suggested that the catalytic activity of DncV diminishes the *csgD* mRNA steady-state level and consequently the rdar morphotype, thus establishing cGAMP as a second messenger in *csgD*-mediated rdar biofilm formation.

### *dncV* downregulates motility by interfering with expression of flagellum regulon genes.

**FIG 3:**
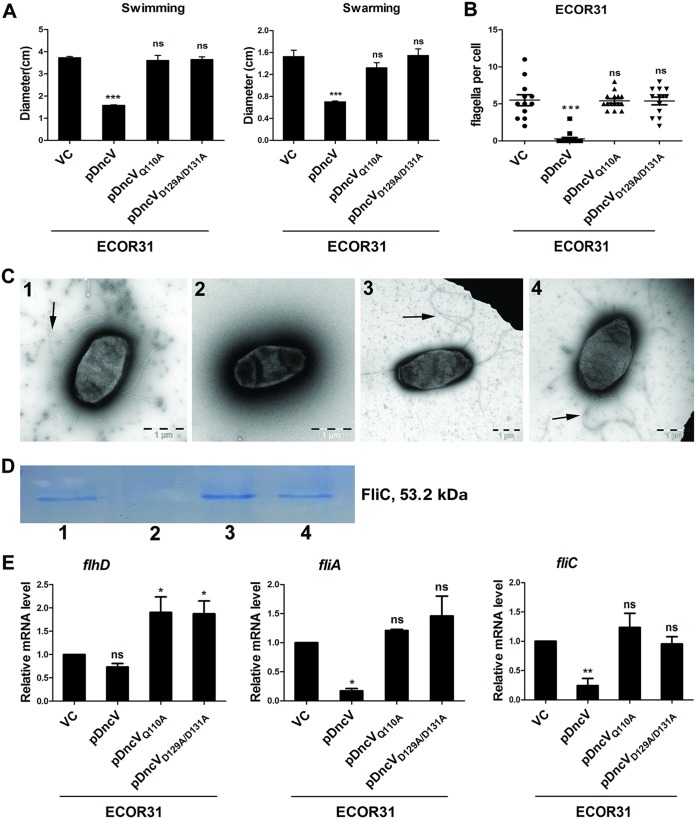
*dncV* downregulates motility in ECOR31 downstream of transcription of the E. coli master regulator FlhD_4_C_2_ of the class 1 flagellum regulon. (A) Flagellum-dependent swimming and swarming motility of wild-type ECOR31 and upon overexpression of DncV and its catalytic mutants DncV_Q110A_ and DncV_D129A/D131A_. Data represent the swimming diameter of cells cultured in LB plates solidified with 0.25% agar for 5 h at 37°C and the swarming diameter of cells cultured on agar plates with 0.8% nutrient broth, 0.5% glucose, and 0.5% Eiken agar after 22 h at 37°C. 1, ECOR31 VC; 2, ECOR31 pDncV; 3, ECOR31 pDncV_Q110A_; 4, ECOR31 pDncV_D129A/D131A_ (VC, vector control pBAD28). (B) Quantification of the number of flagella per cell upon overexpression of *dncV* after visualization by transmission electron microscopy. Number of evaluated cells *n* = 12. Cells were grown at 37°C in LB medium until an OD_600_ = 0.8 to 1 was reached. (C) Flagellin production (arrows) on a representative cell of strain ECOR31 and upon overexpression of *dncV* and its catalytic mutants as observed by transmission electron microscopy. 1, ECOR31 VC; 2, ECOR31 pDncV; 3, ECOR31 pDncV_Q110A_; 4, ECOR31 pDncV_D129A/D131A_ (VC, pBAD28; pDncV, pDncV_Q110A_; pDncV_D129A/D131A_, DncV, DncV_Q110A_, and DncV_D129A/D131A_ cloned in pBAD28). (D) Surface-associated flagellin production was downregulated upon overexpression of *dncV* but not upon overexpression of catalytic mutants. Colloidal Coomassie staining of flagellin extracted from E. coli ECOR31 and its *dncV* derivatives is shown. 1, ECOR31 VC; 2, ECOR31 pDncV; 3, ECOR31 pDncV_Q110A_; 4, ECOR31 pDncV_D129A/D131A_ (VC, pBAD28; pDncV, pDncV_Q110_;, pDncV_D129A/D131A_, DncV, DncV_Q110A_, and DncV_D129A/D131A_ cloned in pBAD28). (E) *dncV* overexpression affects the flagellum regulon cascade downstream of *flhD* transcription. Relative steady-state mRNA levels of class 1 flagellum regulon gene *flhD*, class 2 flagellum regulon gene *fliA*, and class 3 flagellum regulon gene *fliC* as estimated by qRT-PCR are indicated. RNA was isolated from cells grown in LB medium harvested at an OD_600_ of 0.8 to 1 at 37°C and reverse transcribed for qRT-PCR. VC, pBAD28; pDncV, pDncV_Q110A_; pDncV_D129A/D131A_, DncV, DncV_Q110A_, and DncV_D129A/D131A_ cloned in pBAD28. Bars represent the mean values, with error bars representing SD. Differences between mean values were assessed by an unpaired two-tailed Student's *t* test (ns, not significant; *, *P < *0.05; **, *P < *0.01; ***, *P < *0.001 [compared to ECOR31 vector control]).

Cyclic di-GMP inversely coordinates regulation of flagellum-mediated motility with biofilm formation in E. coli (6, 46). We thus wondered whether DncV has a role in motility in ECOR31. Indeed, overexpression of *dncV*_ECOR31_ in E. coli ECOR31 significantly inhibited swimming and swarming motility at 37°C and 28°C (Fig. 3A, Fig. S5A, and data not shown). In contrast, catalytic mutants DncV_Q110A_ and DncV_D129A/D131A_ had no effect compared with the ECOR31 wild-type (Fig. 3A; see also Fig. S5A). Consistent with this result, a chromosomal deletion of *dncV* incrementally, but significantly, enhanced swimming and swarming motility compared to the ECOR31 wild-type (Fig. S5B and C). Of note, in V. cholerae biovar El Tor, expression of *dncV* affects chemotaxis but has no effect on motility and biofilm formation (3). To collect evidence that, indeed, cGAMP and not c-di-GMP produced by DncV inhibits motility, we coexpressed the motility-specific c-di-GMP PDE YhjH. Motility, however, could not be relieved by co-overexpression of YhjH (Fig. S5D).

10.1128/mBio.02492-18.5 FIG S5 Assessment of swimming and swarming motility in E. coli ECOR31 and *dncV* derivatives. (A to C) Swimming and swarming motility upon *dncV* overexpression (A) and swimming (B) and swarming (C) motility upon *dncV* deletion in the E. coli ECOR31 background. Overnight cultures were resuspended to the same cell density (OD_600_ = 5), and 3-µl volumes were inoculated into soft agar plates containing 1% tryptone, 0.5% NaCl, and 0.25% agar (swimming). Swimming diameters were measured after 7 h at 28°C and 5 h at 37°C. Swarming diameters were measured after 12 h (A) or 17 h (C) at 28°C and 37°C. (A) 1, ECOR31 VC; 2, ECOR31 pDncV; 3, ECOR31 pDncV_Q110A_; 4, ECOR31 pDncV_D129A/D131A_; 5, MAE108 VC. VC, pBAD28. pDncV, pDncV_Q110A_, pDncV_D129/131A_ = DncV, DncV_Q110A_, and DncV_D129A/D131A_ cloned in pBAD28. (D) Co-overexpression of DncV and the motility-specific c-di-GMP PDE YhjH. Swimming motility was assessed after 7 h at 28°C and 5 h at 37°C. pDncV, DncV cloned in pBAD28; pYhjH, YhjH cloned in pSRKGm. VC1, pBAD28 and pSRKGm; VC2, pSRKGm. Mean values represent results from three experiments performed with three technical replicates, and error bars represent SD. ns, not significant; *, *P < *0.05; ***, *P < *0.001 (compared to ECOR31 VC; two-tailed Student’s *t* test). Download FIG S5, TIF file, 2.9 MB. Copyright © 2019 Li et al. 2019 Li et al. This content is distributed under the terms of the Creative Commons Attribution 4.0 International license.

Having established the effect of *dncV* on motility as assessed by the agar plate assay (which actually monitors both chemotaxis and motility), we aimed to characterize the level on which DncV_ECOR31_ affects motility. We first addressed flagellin biosynthesis as the farthest-downstream readout phenotype and thus assessed production of cell-associated flagellin upon overexpression of *dncV*. Visualization of intact flagella by TEM showed that overexpression of *dncV* dramatically reduced the total number of flagellum-producing cells as well as the number of flagella per cell, while the two catalytic mutants of DncV had no effect compared with wild-type ECOR31 vector control (Fig. 3B and C). To confirm this result by another assay, we isolated the cell-associated extracellular flagellin, which grossly corresponds to polymerized flagellar filaments (8). While wild-type ECOR31 clearly showed flagellin production, overexpression of DncV, but not overexpression of its catalytic mutants DncV_Q110A_ and DncV_D129A/D131A_, inhibited flagellin production (Fig. 3D). Flagellin production was consistent with the number of flagella as visualized by TEM (Fig. 3B and C). Knowing that flagellum expression was abolished, we assessed the level on which *dncV* affected flagellar regulon genes. Flagellar regulon genes are divided into three classes (Fig. S1A). One gene from each class was chosen for assessment of mRNA steady-state levels of cells grown to the logarithmic phase in LB medium, reflecting motility conditions, by qRT-PCR. The level of the mRNA for *flhD* encoding the FlhD subunit of the class 1 major flagellum regulator FlhD_4_C_2_ was not altered upon overexpression of *dncV* (Fig. 3E). On the other hand, mRNA expression of class 2 and class 3 regulon genes represented by *fliA* encoding the flagellin sigma factor and *fliC* encoding the flagellin subunit, respectively, was significantly diminished upon DncV overexpression (Fig. 3E). In summary, these findings indicate that DncV inhibits motility posttranscriptional of class 1 *flhDC* genes.

### DncV specifically synthesizes 3′3′-cGAMP *in vitro*.

**FIG 4:**
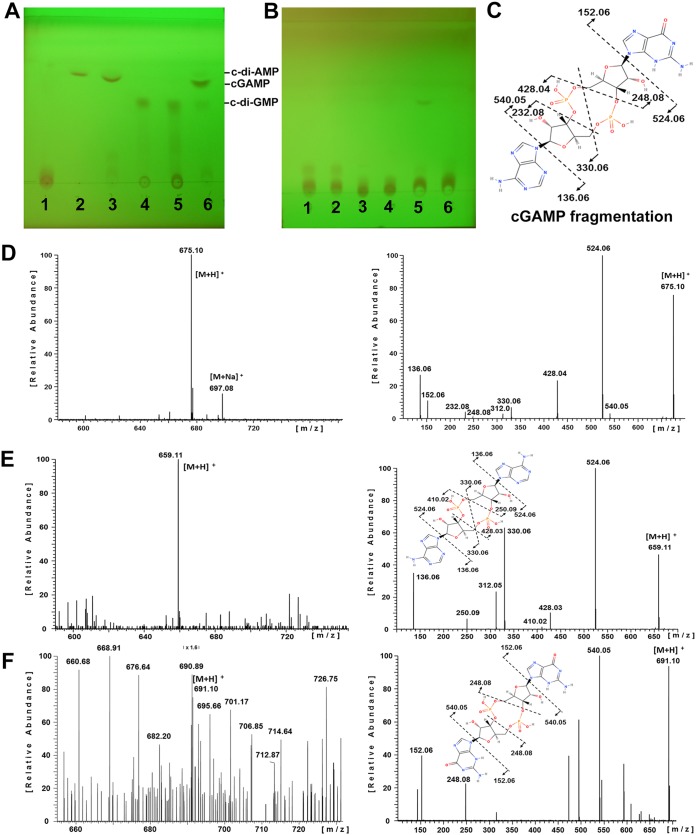
Characterization of the enzymatic activity of DncV by thin-layer chromatography (TLC) and mass spectrometry showed that DncV produces cGAMP, c-di-GMP, and c-di-AMP *in vitro*. (A) TLC analysis of DncV reaction products. 1, ATP + GTP nucleoside triphosphate substrate control; 2, c-di-AMP; 3, ATP + DncV; 4, c-di-GMP; 5, GTP + DncV; 6, ATP + GTP + DncV. (B) TLC analysis showed that DncV_Q110A_ and DncV_D129A/D131A_ are catalytically nonactive. 1, ATP + DncV_Q110A_; 2, ATP + DncV_D129A/D131A_; 3, GTP + DncV_Q110A_; 4, GTP + DncV_D129A/D131A_; 5, ATP + GTP + DncV_Q110A_; 6, ATP + GTP + DncV_D129A/D131A_. DncV (5 µM) was incubated with 5 mM ATP, 5 mM GTP, or 2.5 mM ATP + GTP for 1.5 h at 37°C. Products were run on a TLC plate for 30 min at room temperature. (C) Chemical structure of 3′3′-cGAMP drawn with MolView (freeware created by Herman Bergwerf). Dotted lines inserted into the chemical structure explain the detected fragment ions observed by ESI-MS/MS depicted in panel D. (D) The ESI mass spectrum (positive-ion mode, M + H^+^ = 675.10 Da) of the major enzymatic product from ATP + GTP + DncV identified as cGAMP (left) with the corresponding daughter ion spectrum (MS/MS) (right). (E and F) Analogous results were obtained from c-di-AMP (M + H^+^ = 659.11 Da) (E) and c-di-GMP (M + H^+^ = 691.10 Da) (F). The inserted chemical structures explain the detected fragment ions observed by ESI-MS/MS (right).

Previous studies in V. cholerae characterized DncV as a cDN cyclase that generates cGAMP as the major product (3). To assess the catalytic activity of DncV_ECOR31_, we cloned the open reading frame of *dncV* in an expression vector and subsequently expressed and purified DncV. Evaluation of the enzymatic activity of DncV by the use of the four nucleotides GTP, ATP, CTP, and UTP in all possible combinations was performed *in vitro*, and products were analyzed on thin-layer chromatography (TLC) plates. In the sole presence of ATP and in the sole presence of GTP, DncV synthesized products that ran with the same retention factor as the c-di-AMP and c-di-GMP standards, respectively. In the presence of ATP plus GTP, DncV synthesized a major product with a retention factor between the c-di-AMP and c-di-GMP standards as well as minor products that corresponded to the c-di-GMP and c-di-AMP standards (Fig. 4A). To identify the major product of the enzymatic reaction, the respective bands were cut from the plate and analyzed by mass spectrometry (MS) and tandem mass spectrometry (MS/MS) in the positive-ion mode. The MS result showed that, upon incubation with ATP plus GTP, the major product showed a strong signal of a mass/ion ratio of 675.1 corresponding to a positively charged cGAMP molecule; in addition, fragmentation yielded the expected products (Fig. 4D). Similarly, under conditions of incubation with only ATP and with only GTP, DncV synthesized products that corresponded to positively charged c-di-AMP and c-di-GMP, respectively, with mass/ion ratios of 659.1 and 690.7, as expected (Fig. 4E and F). Thus, similarly to the results seen with DncV_Vcholerae_ (3), analysis of the catalytic activity showed that DncV_ECOR31_ synthesizes three cDNs *in vitro*, with cGAMP as the predominant product. The TLC/MS analysis data were subsequently supported by results obtained by liquid chromatography-tandem mass spectrometry (LC-MS/MS) analysis (Fig. S6A).

10.1128/mBio.02492-18.6 FIG S6 Characterization of enzymatic products and substrate specificity of the dinucleotide cyclase DncV. (A) Analysis of enzymatic reaction products of DncV plus GTP plus ATP by LC-MS/MS. The upper panels show the LC-MS/MS signals of the internal standards of c-di-AMP (^13^C^15^N-c-di-AMP; *m*/*z*: 345 → 146) and c-di-GMP (^13^C^15^N-c-di-GMP; *m*/*z*: 361 → 162) as well as a cGAMP standard (*m*/*z*: 338 → 152). The lower panels show the corresponding analyte signals of c-di-AMP (*m*/*z*: 330 → 136), c-di-GMP (*m*/*z*: 346 → 152), and cGAMP (*m*/*z*: 338 → 152). DncV preferentially uses purine nucleotides as substrates (B to D) and produced 3′3′-c-di-AMP (E and F) and 3′3′-c-di-GMP (E and G) *in vitro*. (B to D) DncV (5 µM per reaction mixture) was incubated with a 5 mM concentration of CTP, UTP, ATP, or GTP or a 2.5 mM concentration of GTP + CTP, GTP + UTP, ATP + CTP, ATP + UTP, or CTP + UTP or a 1.25 mM concentration of ATP + GTP + CTP + UTP for 1.5 h at 37°C. (D to F) DncV (5 µM per reaction mixture) was incubated with a 5 mM concentration of ATP or GTP for 1.5 h at 37°C. After addition of 1 µl of either enzyme RNase T1, S1 nuclease, or RNase T2, incubation was prolonged for another 1 h at 37°C. Reaction products were run on a TLC plate for 30 min at room temperature. (B) 1, ATP + GTP + CTP + UTP; 2, ATP + DncV; 3, CTP + DncV; 4, UTP + DncV; 5, CTP + UTP + DncV; 6, ATP + GTP + CTP + UTP + DncV. (C) 1, ATP + GTP + CTP + UTP; 2, ATP + DncV; 3, ATP + CTP + DncV; 4, ATP + UTP + DncV; 5, CTP + DncV; 6, ATP + GTP + DncV. (D) 1, ATP + GTP + CTP + UTP; 2, GTP + DncV; 3, GTP + CTP + DncV; 4, GTP + UTP + DncV; 5, ATP + GTP + CTP + UTP + DncV; 6, ATP + GTP + DncV. (E to G) 1, ATP; 2, c-di-AMP; 3, ATP + DncV; 4, GTP; 5, c-di-GMP; 6, GTP + DncV. pG = 5′-GMP, Gp = 3′-GMP, pA = 5′-AMP, Ap = 3′-AMP. * = uncharacterized product. Download FIG S6, TIF file, 3.0 MB. Copyright © 2019 Li et al. 2019 Li et al. This content is distributed under the terms of the Creative Commons Attribution 4.0 International license.

The enzymatic activity of the two DncV catalytic mutants was also assessed by running the reaction products on a TLC plate. Incubation of ATP plus GTP with mutant DncV_Q110A_ resulted in a very weak band corresponding to cGAMP, suggesting residual catalytic activity of DncV_Q110A_ in congruence with the phenotypic assessment (Fig. 4B). However, upon incubation of ATP plus GTP with the double mutant DncV_D129A/D131A_, no product was observed, suggesting that this mutant had lost its enzymatic activity (Fig. 4B), again in agreement with the phenotypic analysis. Taken together, these results indicate that the enzymatic activity of DncV is partially dependent on glutamine 110 and absolutely dependent on aspartic acid 129 in combination with aspartic acid 131.

We further assessed the enzymatic activity of wild-type DncV against pyrimidine nucleotides, also in combination with purine nucleotides as substrates. When wild-type DncV was incubated with UTP alone or CTP plus UTP, no product was observed. Interestingly, under conditions of incubation with CTP alone or GTP plus CTP, a weak band running between c-di-AMP and c-di-GMP was observed (Fig. S6B, C, and D), suggesting that DncV can use CTP as the substrate to some extent. Thus, we report these unidentified bands as novel products (Fig. S6B and D), indicating that DncV can synthesize additional products to a minor extent. A previous report which assessed the enzymatic activity of DncV_Vcholerae_ showed that DncV uses ATP and/or GTP as a substrate(s) (3).

**FIG 5:**
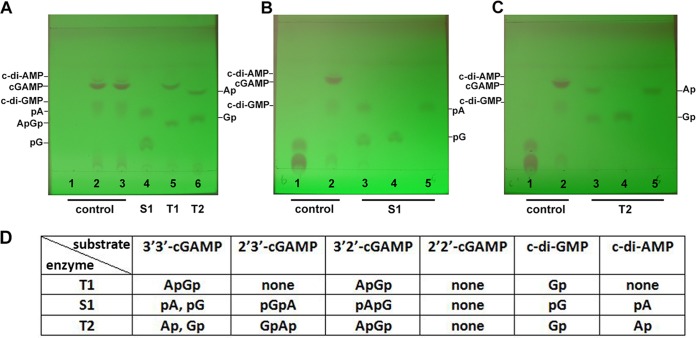
Structural characterization of the DncV reaction product demonstrated a 3′ 3′-linked cGAMP. Digestion of DncV-produced cGAMP with S1 nuclease, RNase T1, and RNase T2 was performed. DncV (5 µM) was incubated with 5 mM ATP + GTP, ATP, or GTP for 1.5 h at 37°C as indicated under A–C. Subsequently, 1 µl of RNase T1, S1 nuclease, or RNase T2 was added for 1 h at 37°C. Products were run on a TLC plate for 30 min at room temperature. (A) 1, ATP + GTP nucleoside triphosphate substrate control; 2, ATP + GTP + DncV; 3, ATP + GTP + DncV at 37°C for 1.5 h; 4, ATP + GTP + DncV/S1 nuclease at 37°C for 1.5 h; 5, ATP + GTP + DncV/RNase T1 at 37°C for 1.5 h; 6, ATP + GTP + DncV/RNase T2 at 37°C for 1.5 h. (B) 1, ATP + GTP trinucleotide control; 2, ATP + GTP + DncV; 3, ATP + GTP + DncV/S1 nuclease; 4, c-di-GMP + S1 nuclease; 5, c-di-AMP + S1 nuclease. (C) 1, ATP + GTP; 2, ATP + GTP + DncV; 3, ATP + GTP + DncV/RNase T2; 4, c-di-GMP + RNase T2; 5, c-di-AMP + RNase T2. (D) Expected enzymatic products of cGAMP isomers upon digestion with the RNase enzymes RNase T1 (T1), S1 nuclease (S1), and RNase T2 (T2). pG = 5′-GMP, Gp = 3′-GMP, pA = 5′-AMP, Ap = 3′-AMP.

As mass spectrometry analysis cannot discriminate between 3′,5′ and 2′,5′ phosphodiester bonds in cyclic dinucleotide molecules, the enzymatic products of DncV were digested with RNase T1, S1 nuclease, or RNase T2 to characterize the structure of *in vitro*-synthesized cGAMP. RNase T1 specifically catalyzes the endonucleolytic cleavage of 3′,5′-phosphodiester bonds only after guanosine (47). The results showed that cGAMP, the major enzymatic product of DncV, was cleaved into a linear dinucleotide by RNase T1 (Fig. 5A), which indicates the presence of one 3′,5′-phosphodiester bond after guanosine. RNase T2 and S1 nuclease both hydrolyze the 3′,5′-internucleotide linkage without base specificity between ribonucleotides but show 5′-phosphomonoesterase and 3′-phosphomonoesterase activity, respectively, while oligomers with 2′,5′ internucleotide linkage are resistant to these nucleases (15, 48, 49). Each enzyme hydrolyzed *in vitro*-synthesized cGAMP into two distinct mononucleotides (Fig. 5A), which suggests the presence of two 3′,5′ phosphodiester bonds in the molecule. Moreover, on TLC plates, these mononucleotides showed the same retention factor as products from the digestion of 3′ 3′-c-di-GMP and 3′ 3′-c-di-AMP with S1 nuclease and RNase T2 (Fig. 5B and C). These analyses are in agreement with the theoretical digestion products of 3′ 3′-cGAMP (compared to 3′ 3′-c-di-GMP and 3′ 3′-c-di-AMP) seen with RNase T1, S1 nuclease, and RNase T2 (Fig. 5D). Taken together, these results demonstrate that DncV_ECOR31_ synthesizes 3′ 3′-cGAMP as a product. Subsequently, we also characterized c-di-AMP and c-di-GMP synthesized by DncV ECOR31 *in vitro* by S1 nuclease, RNase T1, and RNase T2 digestion. The digestion products showed the same running pattern as digestion products from c-di-AMP and c-di-GMP standards (Fig. S6E, F, and G), demonstrating that DncV produced 3′ 3′-c-di-AMP and 3′ 3′-c-di-GMP as by-products.

### DncV promotes biofilm formation in liquid culture.

**FIG 6:**
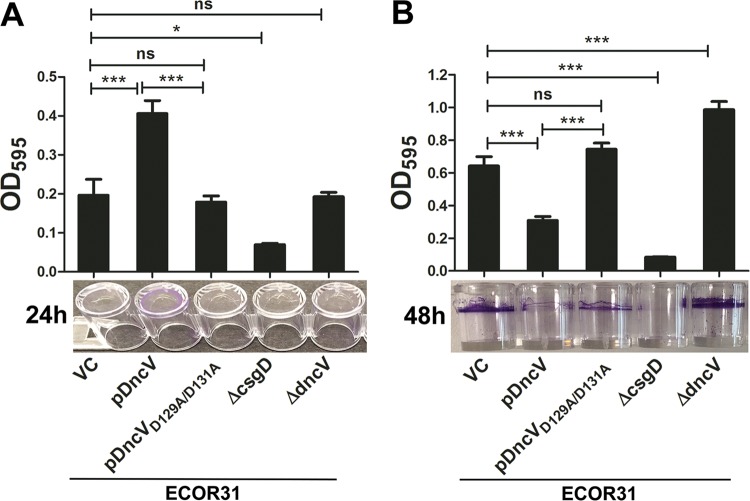
Differential effect of *dncV* on wall-of-well biofilm in E. coli ECOR31. (A and B) Biofilm formation on the 96-well plate polystyrene surface after 24 h (A) and 48 h (B) of incubation at 28°C under steady-state conditions in LB medium. Adherent cells were stained with 0.2% crystal violet, the spatial distributions of biofilm formation in the well was documented (lower panel), and amounts of biofilm were quantified after dissolution in 30% acetic acid at OD_595_. VC, vector control pBAD28; pDncV, pDncV_Q110A_ and pDncV_D129A/D131A_ cloned in pBAD28; DncV, DncV_Q110A_, DncV_D129A/D131A_ cloned in pBAD28. Bars represent the mean values, with error bars representing SD. Differences between mean values were assessed by an unpaired two-tailed Student's *t* test (ns, not significant; *, *P < *0.05; ***, *P < *0.001 [compared to ECOR31 vector control]).

As DncV_ECOR31_ downregulates the *csgD*-mediated rdar colony morphotype on agar plates as well as cell aggregation in liquid medium, we wondered whether DncV could affect alternative biofilm modes such as biofilm formation on abiotic surfaces. To this end, we assessed the effect of *dncV* overexpression on biofilm formation in the 96-well plate assay. Interestingly, we observed a significantly higher level of adherence to the abiotic polystyrene wall of the well surface when wild-type *dncV* was overexpressed from pBAD28 in salt-free LB, LB, and M9 medium after 24 h of incubation in standing culture at 28°C, whereas the deletion mutant had no effect (Fig. 6A; see also Fig. S7A and B). After 48 h of incubation in LB medium, though, overexpression of *dncV* significantly downregulated cell adherence to the abiotic surface, while biofilm formation was upregulated in the *dncV* deletion mutant (Fig. 6B). Investigation of the adherence pattern of cells showed that the 24-h biofilm occurred mainly at the bottom of the well whereas the 48-h biofilm was formed as a ring at the air-liquid interface (Fig. 6, lower panels). The complex effect of *dncV* on biofilm formation might have been caused by promiscuous dinucleotide cyclase activity of DncV *in vivo* or by cGAMP to inversely regulate various adhesive components over time. Indeed, although biofilm formation was *csgD* dependent at 24 and 48 h, *dncV* expression seems to affect adhesins differently at different stages of biofilm development (50).

10.1128/mBio.02492-18.7 FIG S7 Effect of DncV_ECOR31_ on biofilm formation in liquid culture of salt-free LB (A) and M9 (B) medium and DncV_Vcholerae_ on multicellular behavior (C) and swimming motility (D) of E. coli ECOR31. (A-B) *dncV*_ECOR31_ overexpression up-regulates biofilm formation of ECOR31 in liquid culture of salt-free LB (A) and M9 (B) medium. Biofilm formation was assessed by incubating bacteria in 96-well plates in salt-free LB and M9 medium at 28°C for 24 h. Adherent cells were stained with 0.2% crystal violet and dissolved in 30% acetic acid, and absorbance was measured at 595 nm for biofilm quantification. VC, pBAD28; pDncV, pDncV_Q110A_; pDncV_D129A/D131A_, DncV, DncV_Q110A_, and DncV_D129A/D131A_ cloned in pBAD28. Mean values represent results from three experiments performed with six technical replicates, and error bars represent SD. ns, not significant; *, *P < *0.05; **, *P < *0.01; (compared to ECOR31 VC; two-tailed Student’s *t* test). (C and D) Overexpression of DncV_Vcholerae_ consistently downregulated the CsgD-dependent rdar morphotype and swimming motility of ECOR31. These effects were far less pronounced than with *dncV*_ECOR31_. VC, pBAD28. pDncV_ECOR31_, pDncV_Vcholerae_ = pDncV_ECOR31_ and pDncV_Vcholerae_ cloned in pBAD28. Mean values represent results from three experiments performed with three technical replicates, and error bars represent SD. ns, not significant; ***, *P < *0.001 (compared to ECOR31 VC; two-tailed Student’s *t* test). Download FIG S7, TIF file, 3.0 MB. Copyright © 2019 Li et al. 2019 Li et al. This content is distributed under the terms of the Creative Commons Attribution 4.0 International license.

### Complementation of the *dncV*::*Gm* mutant requires multiple gene products.

To this end, we wondered whether the phenotypes of the *dncV*::*Gm* mutant were capable of being complemented by plasmid-derived *dncV*. Of note, DncV expressed from plasmid pBAD28 did not complement the rdar phenotypes at 28°C and 37°C (Fig. S4). Although the gentamicin open reading frame (ORF) has been inserted in parallel to the *dncV* ORF, the insertion might be polar or might disrupt an unrecognized open reading frame. Next, we assessed the effect of gene products cotransferred with *dncV* from V. cholerae VSP-1. Surprisingly, at 28°C, expression of V. cholerae *vc0178* (*capV*)*-dncV* partially downregulated the rdar morphotype, but expression of the putative four-gene operon *vc0178* (*capV*)*-dncV-vc0180-vc0181* had no effect. This effect was temperature sensitive, though. At 37°C, *vc0178* (*capV*)*-dncV* had no effect on rdar morphotype expression, while the overexpression of the four genes *vc0178* (*capV*)*-dncV-vc0180-vc0181* downregulated the rdar morphotype (Fig. S4A). These results show for the first time a physiological role for *capV* encoding a patatin-like phospholipase (51) and for *vc0180-* and *vc0181*-like genes encoding ubiquitin system homologs (data not shown) (52), but also demonstrate complex temperature-dependent functionality of these gene products.

DncV has solely been assessed in a V. cholerae El Tor strain (3). To investigate whether DncV_Vcholerae_ has an effect on ECOR31 physiology similar to that of DncV_ECOR31_, we cloned *dncV*_Vcholerae_ and investigated its impact on ECOR31 physiology. *dncV_Vcholerae_* downregulated CsgD-dependent rdar morphotype expression and motility, although the effect was less pronounced than with *dncV_ECOR31_* (Fig. S7C and D).

DncV produced at least three nucleotides in *in vitro* assays (Fig. 4) (3). To demonstrate the production of cGAMP *in vivo*, DncV expression was induced for 15 and 30 min in ECOR31 cells grown in LB medium to an optical density (OD_600_) of 1. Indeed, we detected 3′3′-cGAMP upon overexpression of DncV in cell extracts, while in the vector control and upon overexpression of the catalytic mutant DncV_D129A/D131A_, a signal was not detected (Fig. S8A and data not shown). Of note, levels of *in vitro* by-products c-di-GMP and c-di-AMP were below the detection limit in the vector control and equally so upon induction of DncV for 15 and 30 min (Fig. S8B and C). These results highlight the impact of *in vivo* assessments of second messenger production in parallel with *in vitro* assessment of catalytic activities (53).

10.1128/mBio.02492-18.8 FIG S8 *In vivo* amounts of cGAMP (A), c-di-GMP (B), and c-di-AMP (C) upon overexpression of DncV. Data representing overexpression of *dncV* for 15 and 30 min display an increase in intracellular cGAMP levels compared to the vector control (VC) and overexpression of the catalytically inactive DncV_D129A/D131A_ mutant in E. coli ECOR31. Expression of *dncV* was induced by addition of 0.1% l-arabinose at an OD_600_ of 1. The concentrations of cGAMP, c-di-GMP, and c-di-AMP were quantified by LC-MS/MS; data are displayed as absolute amounts in the original cell suspension. Each data point represents the mean ± SD of results from five biological replicates. Download FIG S8, TIF file, 3.0 MB. Copyright © 2019 Li et al. 2019 Li et al. This content is distributed under the terms of the Creative Commons Attribution 4.0 International license.

Tags added to proteins can have a pronounced effect on the degree of functionality of the proteins, including alterations in the suppression level of the rdar biofilm (37). Thus, we assessed whether the C-terminal His tag has a role in the activity of DncV *in vivo*. Indeed, the presence of DncV without a tag did not substantially alter the rdar biofilm morphotype (data not shown).

## DISCUSSION

As in many E. coli strains (6, 46), in the animal commensal ECOR31, the temperature-independent *csgD*-dependent rdar biofilm morphotype is positively regulated whereas motility is negatively regulated by c-di-GMP. In this study, we showed that the recently horizontally transferred diguanylate cyclase DncV, with the major *in vitro* and *in vivo* product 3′3′-cGAMP, has the same physiological targets to negatively regulate rdar biofilm formation and flagellum-mediated swimming and swarming motility in E. coli ECOR31. These findings reveal a potential novel role for cGAMP as a second messenger in the regulation of life style transition and multicellular behavior.

The ubiquitous c-di-GMP signaling network is surprisingly variable in E. coli strains (37, 54), with horizontal gene transfer, premature stop codons, and single-amino-acid exchanges to shape the gene pool and activity of cyclic di-GMP turnover proteins (37, 39, 55). While the rdar morphotype can be partially or entirely independent of *csgD* in commensal strains (32, 33, 56), E. coli ECOR31 displays a conventional *csgD*-dependent rdar morphotype (Fig. 1A and B). The diguanylate cyclase AdrA, a GGDEF domain protein regulated by *csgD* (57), required for activation of cellulose biosynthesis, most likely has a similar functionality in ECOR31.

In bacteria, c-di-GMP is a major regulator of bacterial behavior and physiology affecting not only motility and biofilm formation but also the cell cycle, cell morphology, and a multitude of other physiological processes such as photosynthesis, resistance to abiotic stress, and virulence (23, 58, 59). Physiologically elevated concentrations of c-di-GMP activate rdar biofilm formation and *csgD* expression in *S.* Typhimurium and E. coli on the transcriptional level (26, 46). Consistently, the rdar morphotype of ECOR31 is dependent on the catalytic activity of DGCs and PDEs (Fig. 1C and E; see also Fig. S2B and C in the supplemental material), although the chromosomally encoded DGCs and PDEs involved in regulation of the temperature-independent rdar morphotype still need to be identified.

Surprisingly, recently horizontally transferred DncV, most likely through production of the second messenger 3′3′-cGAMP (3), also targets the rdar biofilm morphotype and *csgD* expression on the mRNA steady-state level as does c-di-GMP signaling in E. coli K-12 (Fig. 2). However, cGAMP regulates *csgD* expression opposite the manner seen with c-di-GMP. Whether c-di-GMP and cGAMP use distinct receptors and signaling pathways to regulate *csgD* needs to be sorted out. The identification of cGAMP-specific aptamers indicates that cGAMP specifically affects diverse physiological processes in a number of deltaproteobacterial species (13). VC0178 (renamed CapV), a patatin-like phospholipase encoded upstream of *dncV*, was previously identified as a receptor for cGAMP in V. cholerae biovar El Tor, altering the composition of phospholipids and mediating growth retardation (51). Although CapV, as a part of the horizontally transferred cGAMP signaling module, is also present upstream of *dncV* in ECOR31, growth retardation upon overexpression of *dncV* has not been observed (Fig. S4D), potentially due to tight regulation of the catalytic activity of CapV. This fact does not exclude the possibility that CapV is a receptor for cGAMP, as, consistent with our data, patatin-like phospholipases, which occur in bacteria, fungi, plants, and animals, including humans, have alternative functions as storage proteins and in lipid remodeling and downstream signaling via their transacylase, phospholipase, and lysophospholipase activities (60–64). The hydrolysis products can subsequently initiate a cascade of downstream signaling events, which, in the case of ECOR31, might lead to downregulation of the rdar biofilm and motility. However, we cannot entirely exclude the possibility that, alternatively, *dncV* inhibits the rdar morphotype and/or motility through alternative receptors for cGAMP, enzymatic activity-dependent protein-protein interactions, off-target activities of cGAMP on c-di-GMP turnover proteins or receptors and/or (although the possibility is remote) through *in vivo*-produced c-di-GMP, c-di-AMP, 2′3′-cGAMP (see below) or the still unidentified *in vitro* product (Fig. S6).

Overexpression of DncV showed a variety of distinct phenotypes, including downregulation of late biofilm formation on abiotic surfaces and dissolution of cell aggregation. On the other hand, overexpression of DncV led to enhanced biofilm formation on the abiotic surface after 24 h of growth with a currently unknown molecular basis (Fig. 6). Results of a mannose-sensitive yeast cell aggregation assay suggested that type 1 fimbriae were not involved (data not shown). However, the differential effects of *dncV* overexpression on biofilm phenotypes indicated that DncV and/or 3′3′-cGAMP can affect biofilm formation in a complex way.

Motility, defined as the ability to actively move in liquid or on surfaces, is a major survival mechanism of most microorganisms (65). Flagellum-based swimming and swarming motility in E. coli and *S.* Typhimurium is inhibited by c-di-GMP on the posttranslational level targeting flagellar motor functionality (66–68). We found that *dncV* downregulated motility in E. coli ECOR31 higher up in the flagellum regulon cascade posttranscriptional of *flhDC* expression, affecting the mRNA steady-state levels of class II *fliA* and class III *fliC* (Fig. 3A and E; see also Fig. S5A). In the flagellum regulon cascade, the class I *flhDC* gene products are a major target of transcriptional-to-posttranslational regulation by a number of global regulatory signals such as growth phase and surface-liquid transition directed by global regulators such as cAMP receptor protein (CRP), small RNAs, and degenerated c-di-GMP turnover proteins (69). This is in contrast to V. cholerae, where overexpression of *dncV* does not affect motility but represses chemotaxis (3, 70).

Consistent with *dncV* overexpression, the *dncV* mutant displayed enhanced motility and biofilm formation on abiotic surfaces after 48 h of development (Fig. 6B and S5). What can be the rationale explaining the finding that chromosomally encoded *dncV* participates in restriction of biofilm formation, as such a function can be readily performed by PDEs (71)? Although individual PDEs can also have distinct functionalities due to specific N-terminal signaling domains and differential levels of regulation, various novel regulators can target the unique protein scaffold of DncV, while the output signal cGAMP, in contrast to c-di-GMP, is uniquely associated with repression of biofilm formation and motility. Furthermore, in combination with the patatin-like phospholipase CapV, DncV might participate in unique intra- and extracellular signaling pathways through the products of phospholipid hydrolysis or, at the extreme, might promote autolysis of individual cells. Also, the horizontally cotransferred gene products downstream of *dncV* have a temperature-dependent modulatory role.

cDN cyclase DncV is capable of producing at least four different cDNs, with the most abundant product being 3′3′-cGAMP (Fig. 4; see also Fig. 5). *In vitro* by-products c-di-GMP and c-di-AMP and, *in vivo*, the occasionally observed by-product 2′3′-cGAMP at >10 nM highlight the impact of *in vivo* and *in vitro* investigations (data not shown) (3). The metazoan cyclic GMP-AMP synthase cGAS, which has low sequence similarity but high structural similarity to DncV, produces the noncanonical 2′3′-cGAMP, but anemone cGAS from the ancient species Nematostella vectensis, which diverged from the human ortholog more than 500 million years ago, produces 3′3′-cGAMP (19, 20). Both bacterial and eukaryotic cGAMPs can target the innate immune adaptor STING and other receptors to trigger a broad physiological response (1, 18, 21).

**FIG 7:**
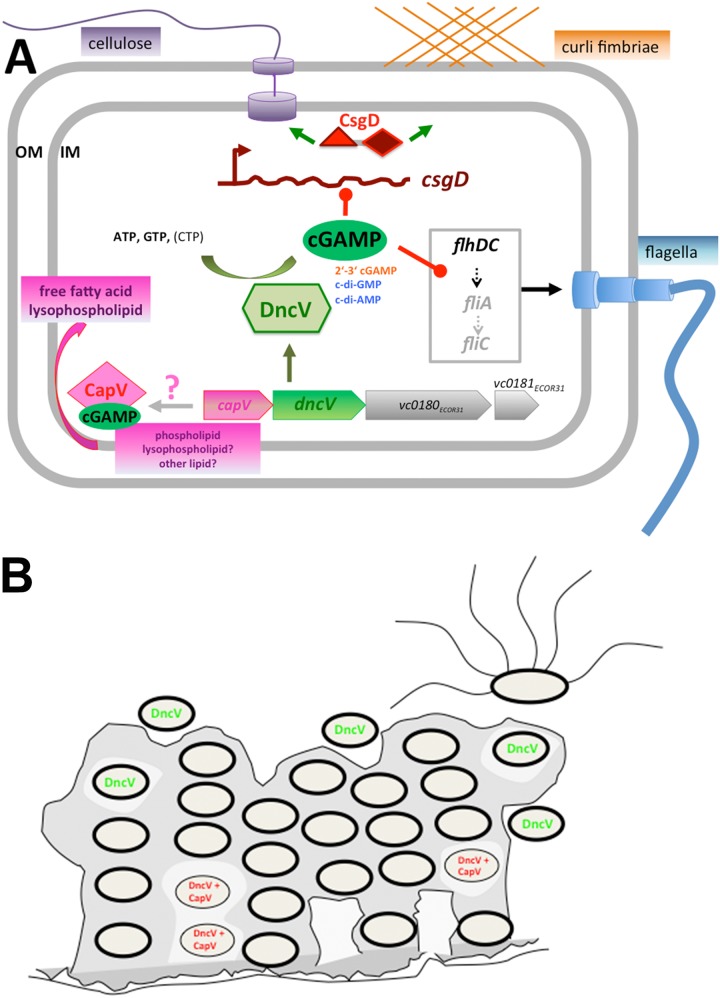
Proposed regulatory role of DncV-produced cGAMP in biofilm formation and motility. (A) Cyclic GAMP is produced by the cyclase DncV. Cyclic GAMP downregulates the steady-state mRNA level of *csgD*, the major activator of the rdar biofilm, as well as amyloid curli fimbriae and the exopolysaccharide cellulose. On the other hand, DncV-produced cGAMP also downregulates mRNA steady-state levels of the flagellin sigma factor FliA class II gene and the class III flagellin subunit FliC gene, thus interfering with flagellum-mediated swimming and swarming motility. *dncV* is located on the horizontally transferred 35-kbp RB-HPI genomic island flanked by V. cholerae VSP-1 homologs of *capV*, *vc0180*, and *vc0181* (3). In V. cholerae, CapV, a patatin-like phospholipase, has demonstrated cGAMP-activated phospholipase activity with subsequent cell lysis upon overexpression (51). Patatin-like phospholipases use phospholipids, lysophospholipids, and potentially other lipids as substrates to produce free fatty acids and lysophospholipids for cell signaling (63). OM, outer membrane; IM, inner membrane. (B) Potential role of DncV in biofilm formation. Expression of *dncV* in biofilms might lead to the downregulation of the extracellular matrix components curli and cellulose with subsequent release of cells from the biofilm. DncV in combination with hyperactivation of CapV might cause autolysis of cells in the biofilm. Note that a role of DncV in biofilm dispersion has not formally been demonstrated.

GGDEF domain diguanylate cyclases are found in over 75% of all bacteria, with the number of genome homologs correlating roughly with genome size. The phylogenetic distribution of the DncV dinucleotide cyclase seems to be restricted to being present in a horizontally transferred single-copy in individual strains rather than in all clones of a species. Surprisingly, though, introduction of the new 3′3′-cGAMP signaling network in E. coli ECOR31 had an immediate effect on the sessility/motility life style transition. However, expression of DncV displayed a phenotype in only a few E. coli strains despite the finding that a patatin-like phospholipase homolog is part of the E. coli core genome (data not shown). This suggests that the strain background, tight repression of enzymatic activity, and/or the presence of cotransferred genes (Fig. S4A) is important for DncV functionality and/or the physiological response. Indeed, under conditions of expression as a stand-alone gene in the ECOR31 wild-type, a strong effect on rdar morphology and motility was observed only with C-terminal His-tagged DncV. A free C terminus, conserved between different DncV homologs (Fig. S3C), might play a functional role, for example, in protein degradation. DncV is horizontally transferred with three additional genes from the VSP-1 island, which are required to complement the *dncV* mutant (Fig. 7; see also Fig. S4A) (3, 30, 51). Besides DncV, a certain class of GGDEF domain proteins has been identified to produce 3′3′-cGAMP (72). Thus, the phylogenetic distribution of cGAMP might be much wider as anticipated as a consequence of the occurrence of close and distantly related dinucleotide cyclase DncV homologs and cGAMP-specific aptamers (3, 13).

*In vitro*, upon incubation with CTP and GTP plus CTP, DncV produces an unidentified compound(s) as a by-product (Fig. S6B, C, and D). Thus, in addition to 3′3′-cGAMP, 2′3′-cGAMP, c-di-GMP, and c-di-AMP, there are other, potentially structurally novel enzymatic products that DncV synthesizes *in vitro* and perhaps even *in vivo*. Although under the conditions of DncV induction (LB medium at an OD_600_ of 1), the cyclic di-GMP concentration was always at the detection limit (as expected since these conditions resemble motility conditions), cGAMP might override *in vivo* c-di-GMP production by direct or indirect manipulation of the activity of diguanylate cyclases or phosphodiesterases. Motility inhibition of DncV is inconsistent with the low c-di-GMP levels (although local effects can never be excluded), but also, lack of motility relieved by the motility-specific PDE YhjH speaks against DncV-mediated elevated c-di-GMP, suggesting that cGAMP, behaving in a direct or indirect manner, or even another alternative cyclic di-nucleotide such as 2′3′-cGAMP is the effective molecule involved in inhibition of motility.

Downregulation of 3′3′-cGAMP concentrations upon prolonged induction of DncV indicated that the cGAMP concentration is tightly regulated, potentially by promiscuous or specific phosphodiesterases in E. coli ECOR31. c-di-GMP is hydrolyzed by proteins with EAL or HD-GYP domains (73, 74). Three HD-GYP domain proteins were identified as 3′3′-cGAMP-specific PDEs in V. cholerae (70). None of these proteins is present in E. coli ECOR31 (data not shown). Overexpression of the major cGAMP PDE from V. cholerae in E. coli ECOR31 and combination with DncV, though, led to inconsistent results, potentially because this enzyme degrades not only cGAMP but also c-di-GMP or other molecules (data not shown). Certainly, more in-depth studies need to be performed to analyze the details of the cGAMP signaling network in E. coli ECOR31. For example, the occurrence of a cGAMP-specific phosphodiesterase activity can be readily tested with cell extracts. Taken together, the results from this work provide an example of the participation of the cGAMP signaling network in regulation of biofilm formation and motility in a commensal E. coli strain, which potentially shows a novel function for cGAMP in the regulation of bacterial physiology.

## MATERIALS AND METHODS

### Bacterial strains and growth conditions.

All strains used in this study are listed in Table S1 in the supplemental material. For cloning, E. coli strains were cultured in Luria-Bertani (LB) liquid media or on solid LB agar plates that were, if relevant, supplemented with antibiotics (100 µg/ml ampicillin; 25 µg/ml chloramphenicol; 30 µg/ml gentamicin) at the indicated temperatures. l-Arabinose (Sigma) (0.1%) and 1 mM isopropyl β-d-1-thiogalactopyranoside (IPTG; Sigma) were used for induction of gene expression.

10.1128/mBio.02492-18.9 TABLE S1 Bacterial strains and plasmids used in this study. Download Table S1, PDF file, 2.6 MB. Copyright © 2019 Li et al. 2019 Li et al. This content is distributed under the terms of the Creative Commons Attribution 4.0 International license.

### Mutant construction.

Construction of chromosomal deletion mutants was performed by homologous recombination (75). In brief, a gentamicin resistance cassette was amplified from pSRKGm by PCR with primers containing 40 nucleotides at the beginning and end of the target gene, including the start and stop codon, and was electroporated into ECOR31 carrying helper vector pSIM7. Primers are listed in Table S2.

10.1128/mBio.02492-18.10 TABLE S2 Primer used in this study. Download Table S2, PDF file, 2.1 MB. Copyright © 2019 Li et al. 2019 Li et al. This content is distributed under the terms of the Creative Commons Attribution 4.0 International license.

Mutants were identified by PCR with primers outside the recombination region and colony purified by streaking at least twice on LB plates with 20 µg/ml gentamicin at 30°C. To cure plasmid pSIM7, the mutants were streaked on LB plates without any antibiotics, incubated for 8 h at 37°C, and subsequently tested for susceptibility to chloramphenicol.

### Plasmid construction.

All plasmids used in this study are listed in Table S1. Genes of interest were amplified by PCR from the ECOR31 chromosome. The PCR products were digested with XbaI/HindIII (NEB) restriction enzymes and ligated into pBAD28 vector using a Rapid DNA ligation kit (Roche Diagnostics). Inserted DNA sequences were confirmed by DNA sequencing.

### Site-directed mutagenesis.

Site-directed mutagenesis was performed using a QuikChange II site-directed mutagenesis kit according to the instructions of the manufacturer (Agilent Technologies). All mutations were confirmed by DNA sequencing.

### Phenotypic assays. (i) rdar colony morphotype.

To visualize the expression of cellulose and curli fimbriae, 5 µl of an overnight culture suspended in water (optical density at 600 nm [OD_600_] = 5) was spotted onto a salt-free LB agar plate containing the dye Congo red (Sigma) (40 µg/ml) and Coomassie brilliant blue G-250 (Sigma) (20 µg/ml) or calcofluor white (Fluorescence Brightener 28; Sigma) (50 µg/ml). Plates were incubated at 28°C or at 37°C. Colonies were photographed at different time points to analyze the development of the colony morphology structure and dye binding. Only colony morphotypes from the same plate were compared.

### (ii) Motility assay.

To observe swimming motility, 3 µl of an overnight culture suspended in water (OD_600_ = 5) was inoculated into soft agar medium containing 1% tryptone, 0.5% NaCl, and 0.25% agar (76). The swimming diameter was measured after 7 h at 28°C and 5 h at 37°C. To observe swarming motility, a single colony was introduced onto the swarming agar medium consisting of 0.8% nutrient broth and 0.5% glucose solidified by 0.5% Eiken agar (77). The swarming diameter was measured after 16 h at 28°C and 22 h at 37°C.

### (iii) Aggregation (clumping) assay.

Cell aggregation (clump formation) was determined by inoculating bacteria from an overnight culture into salt-free LB medium with a starting OD_600_ of 0.01. Aerobic conditions were 10 ml of culture in a 50-ml flask; microaerobic conditions were 60 ml culture in a 100-ml flask with shaking at 180 rpm/min at 28°C and 37°C and with protein induction by 0.1% l-arabinose. Cell aggregation was visually observed throughout the growth phase for up to 24 h with samples collected for detection of CsgD expression by Western blot analysis. After 14 h, samples were collected for RNA isolation.

### (iv) Biofilm formation assay.

Biofilm formation (adherence to the wall of the well) was assessed by incubating bacteria in 96-well plates in LB with or without salt and M9 medium at 28°C for 24 h. Adherent cells were stained with 0.2% crystal violet, washed, and dissolved in 30% acetic acid. The absorbance of dissolved crystal violet was measured at a wavelength of 595 nm.

### CsgA isolation.

Major curli fimbriae subunit CsgA was enriched as previously reported (78). Briefly, 5 mg of bacteria was harvested from the plates and resuspended in Tris-EDTA (TE) buffer containing 10 mM Tris-HCl, 1 mM EDTA (pH 7.5), and 2% SDS. After heating to 95°C for 45 min, the insoluble material was washed three times with distilled water, resuspended in 80 µl 100% formic acid to depolymerize CsgA, and then incubated on ice for 10 min. After removal of the formic acid in a vacuum centrifuge, the cell pellet was resuspended in 25 µl SDS sample buffer and run on a 15% SDS-PAGE gel. Isolated CsgA was visualized by Coomassie blue staining.

### Isolation of cell-associated flagellin.

Bacterial cell-associated flagellin was isolated as described previously (8). Briefly, an agar plate-grown single colony was inoculated in LB medium and cultured overnight at 37°C at 150 rpm. After dilution to an OD_600_ of 0.01, culturing was continued to reach an OD_600_ of 0.6. Flagella were sheared off by pressing the cell suspension 15 times through a syringe with a needle (BD Microlance) of 0.51-mm diameter. A 1-ml volume of samples was collected and centrifuged at 17,000 × *g*, and the supernatant was mixed with cold trichloroacetic acid (Sigma) (3:1 [vol/vol]). Samples were incubated at −20°C for 2 h, followed by centrifugation at 17,000 rpm for 40 min. The cell pellet was collected for SDS-PAGE gel analysis (4% stacking gel, 15% running gel), and separated proteins were stained by colloidal Coomassie brilliant blue.

### Transmission electron microscopy.

Bacterial flagella were visualized by transmission electron microscopy (TEM). Cells from overnight culture were diluted in LB medium and cultured to an OD_600_ of 0.8 to 1, and protein expression was induced by 0.1% l-arabinose. An aliquot of 3 µl from each samples was then added to a grid with a glow-discharged carbon-coated supporting film for 3 min. The excess solution was soaked off by the use of filter paper, and the grid was rinsed by adding 5 µl of distilled water for 10 s. The grid was subsequently stained with 5 µl 1% uranyl acetate (Sigma)–water for 7 s, and the grid was air-dried. The samples were examined in a Hitachi HT 7700 (Hitachi, Tokyo, Japan) electron microscope at 80 kV, and digital images was taken by a Veleta camera (Olympus, Münster, Germany).

### Scanning electron microscopy.

The bacterial cells were fixed in 1.5 ml fixative solution (0.5% glutaraldehyde, 2.5% paraformaldehyde in 10 mM HEPES, pH 7.0) for 2 h at 4°C. The fixed biofilm sample was processed for observation by scanning electron microscopy (SEM), applying dehydration with acetone, critical-point drying, and sputter coating with gold/palladium. Samples were examined in a Zeiss Merlin field emission scanning electron microscope at an acceleration voltage of 5 kV with an Everhart-Thornley secondary emission (SE) detector and an in-lens SE detector at a 30:70, 70:30, or 77:23 ratio.

### RNA isolation and qRT-PCR.

The total bacterial RNA was isolated from cells grown in liquid culture from two different growth phases (from the logarithmic-growth phase at an OD_600_ of 0.8 to 1 and from the stationary-growth phase grown for 14 h) by the hot acid-phenol method (44). After treatment with DNase (Ambion RiboPure-Yeast DNase) was performed, the RNA quality was assessed by gel electrophoresis and PCR. The RNA concentration was measured by the use of a NanoDrop 2000 system (Thermo Scientific). cDNA synthesis was performed using 1 µg RNA and a High-Capacity cDNA reverse transcription kit (Applied Biosystems) according to the manual instructions. Quantitative PCR (qPCR) was run with SYBR green (iTaq universal SYBR green Supermix; Bio-Rad) and performed using a model 7500 real-time PCR system (Applied Biosystems) in the standard mode. Primers are listed in Table S2. The data were analyzed (assuming exponential amplification) using the threshold cycle (2^−ΔΔ^*^CT^*) method (35). The *rpsV* gene was used as an endogenous control for internal normalization.

### Protein purification.

The *dncV* gene was PCR amplified from genomic DNA and was subsequently cloned into pET28a expression vector, which provides a 6× C-terminal His tag sequence, and transformed into E. coli BL21(DE3) cells (Novagen). Cells were cultured in LB medium at 37°C to an OD_600_ of 0.5 to 0.8, 0.5 mM IPTG was added, and the culture was further incubated for 16 h at 18°C. The cells were lysed in lysis buffer (50 mM NaH_2_PO_4_ [pH 8.0], 300 mM NaCl, 20 mM imidazole, 5% glycerol, 2 mM MgCl_2_, 1 mM phenylmethylsulfonyl fluoride, 3 mM 2-mercaptoethanol) by sonication. The supernatant was passed through a nickel-nitrilotriacetic acid (Ni-NTA) (Qiagen) column, and the resin was washed with a 5-bed volume of washing buffer (50 mM NaH_2_PO_4_ [pH 8.0], 300 mM NaCl, 20 mM imidazole, 3 mM 2-mercaptoethanol). The bound proteins were eluted with elution buffer (50 mM NaH_2_PO_4_ [pH 8.0], 300 mM NaCl, 300 mM imidazole, 3 mM 2-mercaptoethanol). The eluted protein was dialyzed against washing buffer using Amicon Ultra-4 filter units (Merck) (30-kDa cutoff) and then stored in aliquots at −80°C. The entire purification process was performed on ice at 4°C.

### Dinucleotide cyclase assay.

The dinucleotide cyclase assay was performed in reaction buffer (50 mM Tris [pH 8.0], 10 mM MgCl_2_, 100 mM NaCl) with the indicated NTPs (5 mM ATP/GTP/CTP/UTP [2.5 mM in cases in which two nucleotides were used]). The reaction was started by adding DncV (Q110A or D129A/D131A) to reach a final concentration of 5 µM, the reaction mixture was incubated at 37°C for 1.5 h, and the reaction stopped by heating at 95°C for 20 min. The samples were subsequently analyzed by thin-layer chromatography (TLC).

To characterize the chemical structure, products of the DncV enzymatic reaction were subsequently incubated with 1 µl of RNase T1 (Thermo Scientific)–100 mM Tris-HCl (pH 7.5)–10 mM EDTA reaction buffer; S1 nuclease (Thermo Scientific)–40 mM sodium acetate (pH 4.5)–300 mM NaCl–2 mM ZnSO_4_ reaction buffer; or RNase T2 (MoBiTec)–125 mM NH_4_Ac (pH 4.5) for 1 h at 37°C.

### Thin-layer chromatography.

Thin-layer chromatography was performed on a 5-by-10-cm silica 60 UV254 TLC plate (Macherey-Nagel). A 2-µl sample from an enzymatic reaction was spotted onto the TLC plate and run for 30 min until the front of the plate was reached with the following running solvent: n-propanol(1-propanol)/ammonium hydroxide/water (11:7:2 [vol/vol/vol]). After the run was performed, the plate was dried in a fume hood and the bands were visualized by exposure to shortwave UV light at 254 nm. The *R_f_* value for each product was calculated.

### Extraction of nucleotides produced *in vivo*.

The extraction of cDNs from bacterial cells was performed as reported previously (79). Briefly, overnight cultures from individual colonies were diluted to an OD_600_ of 0.01 and grown to an OD_600_ of 1 at 37°C in LB medium with 200 rpm shaking. Expression of *dncV* was induced by the addition of 0.1% l-arabinose for 15 min or 30 min. A 3-ml volume of the cell suspension was pelleted and resuspended in 500 µl of ice-cold extraction solvent (acetonitrile/methanol/water/formic acid, 2/2/1/0.02 [vol/vol/vol/vol]), followed by boiling for 10 min. Three subsequent extracts were combined and frozen at −20°C overnight. The extract were centrifuged for 10 min at 20,800 × *g*, evaporated to dryness in a Speed-Vac (Savant), and analyzed by LC-MS/MS.

### Mass spectrometry and tandem mass spectrometry.

Products from the enzymatic assay were characterized by MS and MS/MS. Briefly, product bands of samples that had been spotted and run on TLC plates for 30 min were cut out, dissolved in MS-grade water (Sigma), and centrifuged twice at 13,000 rpm for 30 min each time to remove the silica. Supernatant containing 1 µg of enzymatic product was collected and analyzed by MS and electrospray ionization-tandem MS (ESI-MS/MS) in the positive-ion mode (6, 79).

### Western blot analysis.

To detect CsgD expression, a 5-mg (wet weight) volume of bacterial cells from agar plates or liquid culture was placed into 200 µl SDS sample buffer and heated to 95°C for 10 min. The amount of protein was analyzed by Coomassie blue staining after gel separation. Sample volumes containing equal amounts of proteins were separated by SDS-PAGE (4% stacking and 12% resolving gel) and transferred onto a polyvinylidene difluoride (PVDF) membrane (Millipore). The membrane was blocked with 5% skim milk overnight, and the protein was detected with a polyclonal E. coli anti-CsgD peptide antibody (1:5,000 dilution) (56). Horseradish peroxidase-conjugated goat anti-rabbit IgG was used as the secondary antibody (Jackson ImmunoResearch Laboratories Inc.) (1:2,000 dilution). The targeted proteins were visualized using ECL light detection reagent (Roche) and a Luminescent Image Analyzer (LAS-1000plus; Fujifilm).

### Bioinformatic analyses.

A BLAST search against the NCBI protein database was performed with standard parameters using DncV_ECOR31_ as a query and with all distinct protein sequences from E. coli and V. cholerae and a representative sequence from each genus with >40% identity to be selected. Sequences were aligned using ClustalX 2.1 and standard parameters, and a phylogenetic reconstruction was performed using maximum likelihood in MEGA 7.0 (80). The robustness of the tree was tested with 1,000 bootstrap replications. Phylogenetic reconstruction performed with neighborhood joining resulted in an identical tree (not shown). A sequence logo was constructed with WebLogo (81) using aligned sequences (columns with fewer than 2 amino acids were omitted).
