# Supplementary figures and images for: DncV Synthesizes Cyclic GMP-AMP and Regulates Biofilm Formation and Motility in Escherichia coli ECOR31
Source: mBio. 2019 Mar 5;10(2):e02492-18. doi: 10.1128/mBio.02492-18 (PMC6401482; doi:10.1128/mBio.02492-18)

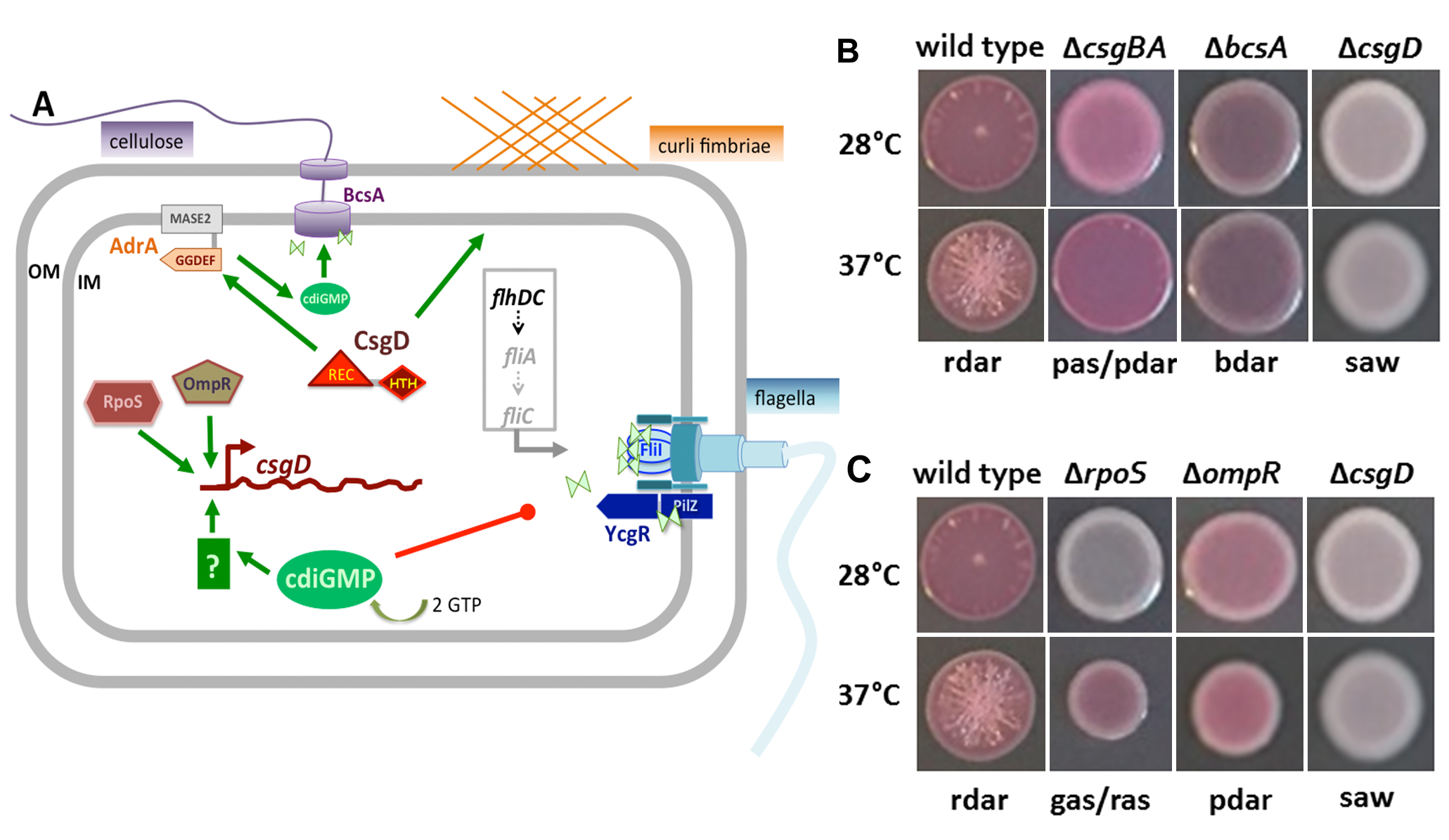

Supplement: FIG S1 [file mBio.02492-18-sf001.tif]

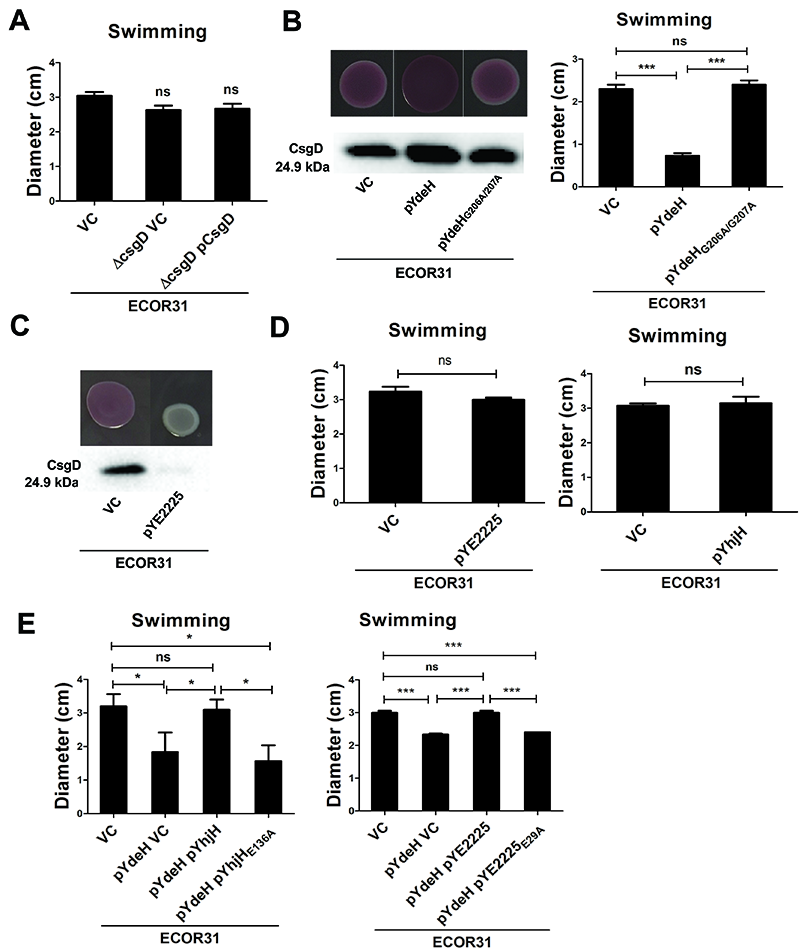

Supplement: FIG S2 [file mBio.02492-18-sf002.tif]

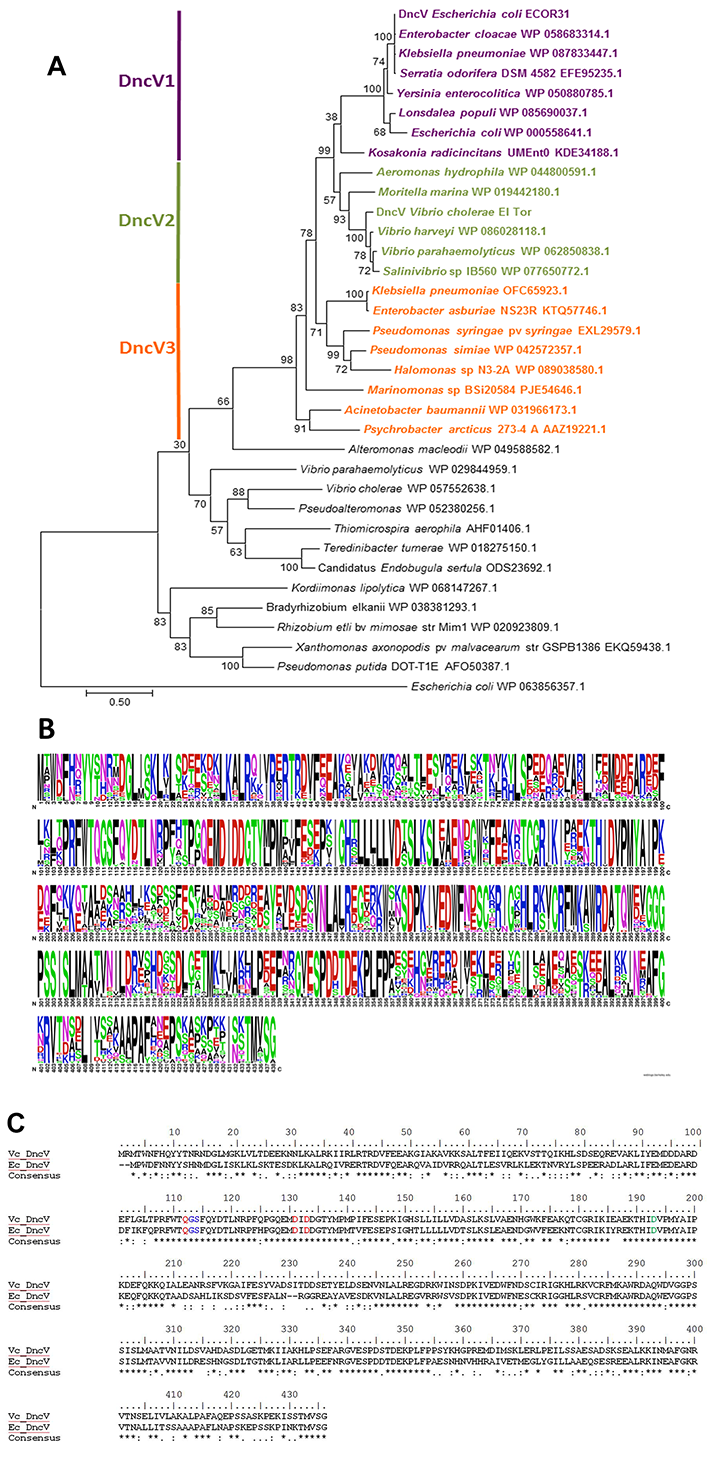

Supplement: FIG S3 [file mBio.02492-18-sf003.tif]

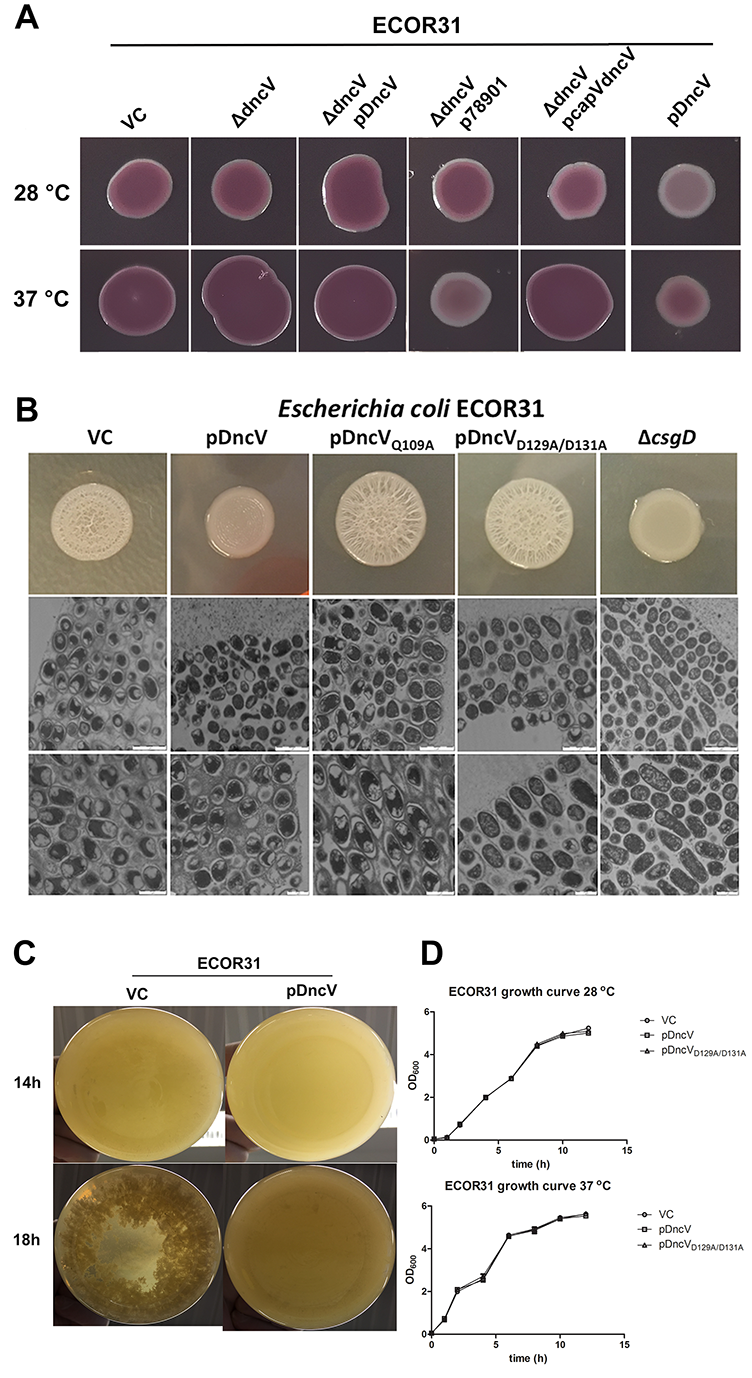

Supplement: FIG S4 [file mBio.02492-18-sf004.tif]

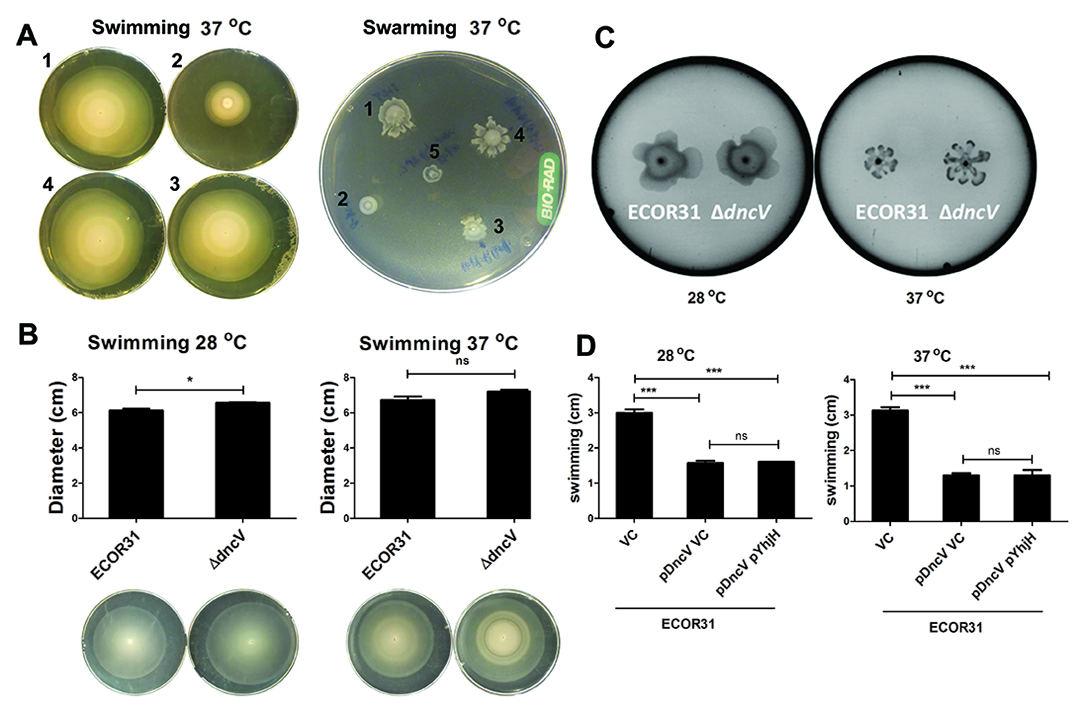

Supplement: FIG S5 [file mBio.02492-18-sf005.tif]

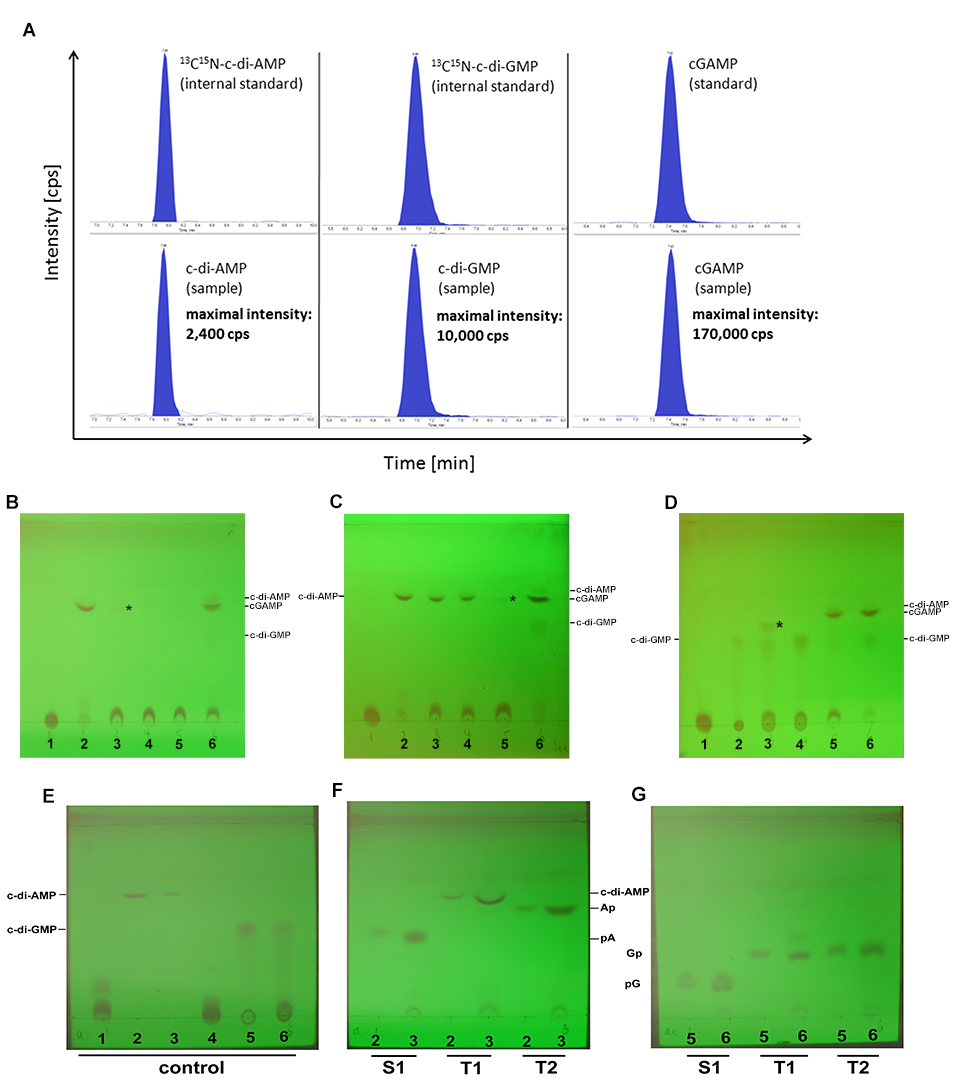

Supplement: FIG S6 [file mBio.02492-18-sf006.tif]

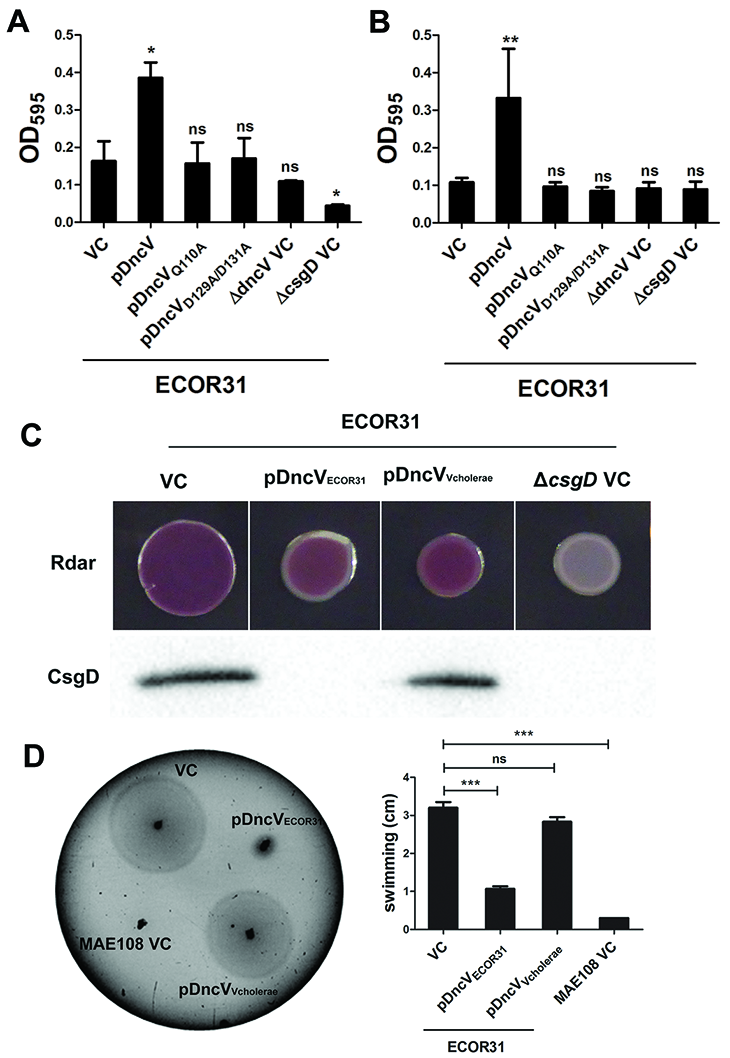

Supplement: FIG S7 [file mBio.02492-18-sf007.tif]

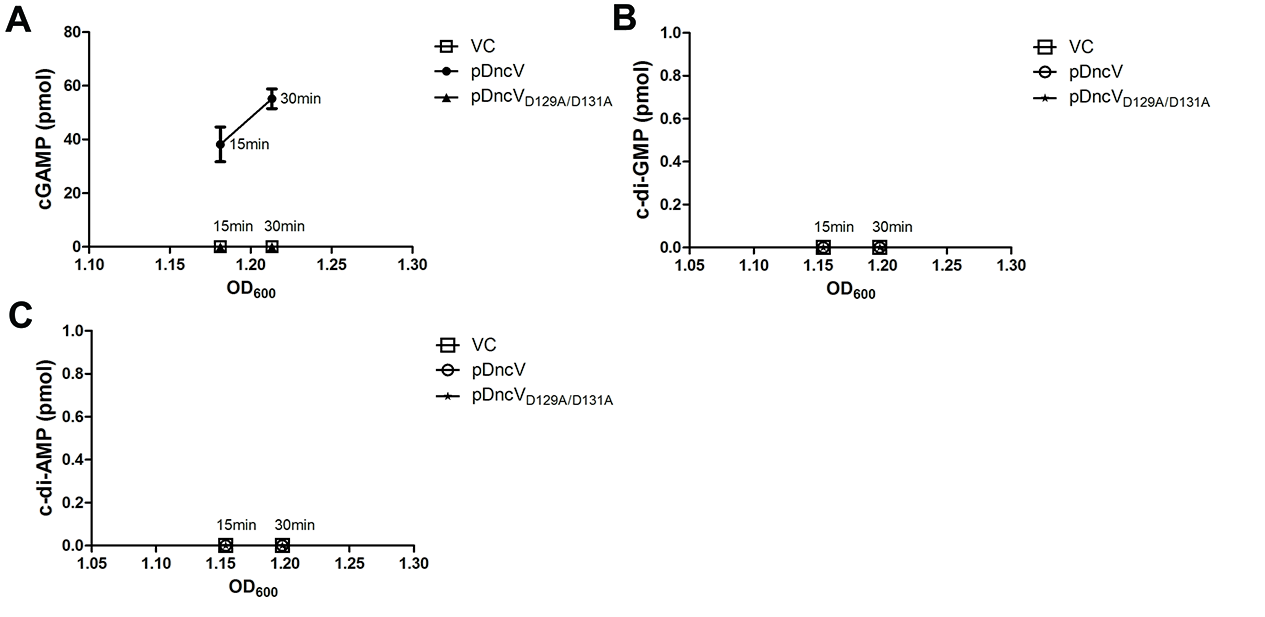

Supplement: FIG S8 [file mBio.02492-18-sf008.tif]
