# Supplementary material for: DncV Synthesizes Cyclic GMP-AMP and Regulates Biofilm Formation and Motility in Escherichia coli ECOR31
Source: mBio. 2019 Mar 5;10(2):e02492-18. doi: 10.1128/mBio.02492-18 (PMC6401482; doi:10.1128/mBio.02492-18)
Supplement: TABLE S1 [file mBio.02492-18-st001.pdf]

Table S1 Bacterial strains and plasmids used in this study

**Strain or plasmid**	**Genotype or description**	**Reference or source**
E. coli strains		
ECOR31	Strain 31 of the *E. coli* reference collection (ECOR); Tc^r, Km^r, Ap^r, Cm^s, Tp^s	(1)
K-12 Top10	F- *mcrA* Δ(*mrr-hsdRMS-mcrBC*) ϕ80*lacZ*ΔM15 Δ*lacX74* *nupG* *recA1* *araD139* Δ(*ara-leu*)7697 *galE15* *galK16* *rpsL*(StrR) *endA1* λ-	Invitrogen
BL21(DE3)	F^- *ompT hsdSB*($r^-_B m^-_B$) *gal dcm* (DE3)	Novagen
Other strains		
Vibrio cholerae C6706	Wild type, El Tor	Laboratory collection
Salmonella typhimurium MAE108	UMR1 Δ*fljB fliC*::Cm^r	(2)
Plasmids		
pBAD28	Arabinose-regulated promoter; Amp^r; Cm^r	(3)
pSIM7	pBBR1-derived, expresses temperature inducible Lambda-Red recombinase system; Cm^r	(4)
pET28a (+)	Expression vector with T7 promoter; Km^r	
pSRKGm	pBBR1MCS-5-derived expression vector containing *lac* promoter and *lacI*^{q}, *lacZ*$^{\alpha+}$; Gm^r	(5)
pDncV	pBAD28::DncV-6xHis; Cm^r, Amp^r	This study
pDncV$_{Q110A}$	pBAD28::DncV$_{Q110A}$-6xHis; Cm^r, Amp^r	This study
pDncV$_{D129A/D131A}$	pBAD28::DncV$_{D129A/D131A}$-6xHis; Cm^r, Amp^r	This study
pCsgD	pBAD28::CsgD; Cm^r, Amp^r	This study
pYdeH	pBAD28::YdeH; Cm^r, Amp^r	(6)
pYdeH$_{G206A/G207A}$	pBAD28::YdeH$_{G206A/G207A;}$ Cm^r, Amp^r	(6)
pAdrA	pBAD28::AdrA; Cm^r, Amp^r	This study, (7)
pAdrA$_{G288A/G289A}$	pBAD28::AdrA$_{G288A/G289A}$; Cm^r, Amp^r	This study, (7)
pYhjH	pSRKGm::YhjH; Gm^r	(8)
pYhjH$_{E136A}$	pSRKGm::YhjH$_{E136A}$; Gm^r	(8)
pYE2225	pSRKGm:: YE2225; Gm^r	(9)
pYE2225$_{E29A}$	pSRKGm::YE2225$_{E29A}$; Gm^r	(9)
pDncV$_{Vcholerae}$	pBAD28::DncV$_{Vcholerae}$	This study
p*capVdncV*	pBAD28::*capV dncV*	This study
p78901	pBAD28::*capV dncV vc0180 vc0181*	This study

References

1. **Ochman H, Selander RK.** 1984. Standard reference strains of Escherichia coli from natural populations. J Bacteriol **157:**690-693.
2. **Rochon M, Römling U.** 2006. Flagellin in combination with curli fimbriae elicits an immune response in the gastrointestinal epithelial cell line HT-29. Microb Infec / Institut Pasteur **8:**2027-2033.
3. **Guzman L-M, Belin D, Carson MJ, Beckwith J.** 1995. Tight regulation, modulation, and high-level expression by vectors containing the arabinose PBAD promoter. J Bacteriol **177:**4121-4130.
4. **Datta S, Costantino N, Court DL.** 2006. A set of recombineering plasmids for gram-negative bacteria. Gene **379:**109-115.
5. **Khan SR, Gaines J, Roop RM, Farrand SK.** 2008. Broad-host-range expression vectors with tightly regulated promoters and their use to examine the influence of TraR and TraM expression on Ti plasmid quorum sensing. Appl Environ Microbiol **74:**5053-5062.
6. **Jonas K, Edwards AN, Simm R, Romeo T, Römling U, Melefors Ö.** 2008. The RNA binding protein CsrA controls cyclic di‐GMP metabolism by directly regulating the expression of GGDEF proteins. Mol Microbiol **70:**236-257.
7. **Simm R, Morr M, Kader A, Nimtz M, Römling U.** 2004. GGDEF and EAL domains inversely regulate cyclic di‐GMP levels and transition from sessility to motility. Mol Microbiol **53:**1123-1134.
8. **Simm R, Lusch A, Kader A, Andersson M, Römling U.** 2007. Role of EAL-containing proteins in multicellular behavior of Salmonella enterica serovar Typhimurium. J Bacteriol **189:**3613-3623.
9. **El Mouali Y, Kim H, Ahmad I, Brauner A, Liu Y, Skurnik M, Galperin MY, Römling U.** 2017. Stand-alone EAL domain proteins form a distinct subclass of EAL proteins involved in regulation of cell motility and biofilm formation in enterobacteria. J Bacteriol **199:**e00179-00117.
