# Supplementary material for: DncV Synthesizes Cyclic GMP-AMP and Regulates Biofilm Formation and Motility in Escherichia coli ECOR31
Source: mBio. 2019 Mar 5;10(2):e02492-18. doi: 10.1128/mBio.02492-18 (PMC6401482; doi:10.1128/mBio.02492-18)
Supplement: TABLE S2 [file mBio.02492-18-st002.pdf]

Table S2 Primer used in this study.

**Primer**	**Sequence (5'-3')**
Gene cloning	
DncV-XbaI-fw	TTTTCTAGA GCAGGAGAAACTCTG ATG CCTTG[a]
DncV-HindIII-rv	TTTAAGCTT TCA ATGATGATGATGATGATGATGGCCACTTACCATCGT[a]
CsgD-XbaI-fw	TGCTCTAGA AAGCGGGGTTTCATCATGTTTAATGAAG[a]
CsgD-SphI-rv	ACATGCATGC GTTTCATGGCTTTATCGCCT GAGG[a]
VC0178-81-<br>SacI-fw	GGCGAGCTC GCTATATTCTCTGGTTATGGGGTTTTCAATGTCTG[a]
VC0178-81-<br>SphI-rv	ACATGCATGC TTACTTCTCT CTTATAC[a]
DncVvc-XbaI-fw	GCTCTAGA AAATCTTAGAGGAGAGTGAGAAT[a]
DncVvc-SphI-rv	ACATGCATGC TCA ATGATGATGATGATGATG GCCACTTAC CATTGTG[a]
Gene deletion	
DncV-ko-FW	ATGCCTTGGGATTTTAACAATTACTATAGTCACAATATGG<br>CGAACCCAGTTGACATAAGC[b]
DncV-ko-RV	TCAGCCACTTACCATCGTTTTGTTGATTGGTTTAGATGAT<br>CAATTTACCG AACAACTCCG CG[b]
DncV-ctrl-Fw	GGATAATGCCACGAAGAGTT
DncV-ctrl-Rv	TAAAATTACCGGCATCAGTC
CsgD-H1P1-fw	ATGTTTAATGAAGTCCATAGTATTCATGGTCATACATTAT<br>CGAACCCAGTTGACATAAGC[b]
CsgD-H2P2-rv	TTATCGCCTGAGGTTATCGTTTGCCCAGGAAACCGCTTGT<br>CAATTTACCG AACAACTCCG CG[b]
CsgD-ctrl-fw	ACACAGCAGTGCAACATCTGTC
CsgD-ctrl-rv	GAATCTTCACGCCGTTGAGG
csgBA-ko-fw	ATGTACGACCAGGTCCAGGGTGACAACATGAAAAACAAAT<br>CGAACCCAGTTGACATAAGC[b]
csgBA-ko-rv	TTAGTACTGA TGAGCGGTCG CGTTGTTACC AAAGCCAACC<br>CAATTTACCGAACAACTCCGCG[b]
csgBA-ctrl-fw	CCGCAGACATACTTTCCATC
bcsA-ko-fw	ATGAGTATCCTGACCCGGTGGTTGCTTATCCCGCCGGTCA<br>CGAACCCAGTTGACATAAGC[b]
bcsA-ko-rv	TCATTGTTGA GCCAAAGCCT GATCCGATGG TTGTGCCGTT<br>CAATTTACCGAACAACTCCGCG[b]
bcsA-ctrl-fw	GCGGCTGAAGAGATATTGAC
ompR-ko-fw	ATGCAAGAGAACTACAAGATTCTGGTGGTCGATGACGACA<br>CGAACCCAGTTGACATAAGC[b]
ompR-ko-rv	TCATGCTTTA GAGCCGTCCG GTACAAAGAC GTAGCCCAGG<br>CAATTTACCGAACAACTCCGCG[b]
ompR-ctrl-fw	CCAGATTTAGCTGGTGACGAACG
rpoS-ko-fw	ATGTTCCGTCAAGGGATCACGGGTAGGAGCCACCTTATGA<br>CGAACCCAGTTGACATAAGC[b]
rpoS-ko-rv	TTATTCGCGG AACAGCGCTT CGATATTCAG CCCCTGCGTT<br>CAATTTACCGAACAACTCCGCG[b]
rpos-ctrl-fw	CCGTAAACCCGCTGCGTTATTTG

Gm-inside-rv	GTCTTACTAC GGAGCAAGTT CC
Site-directed mutagenesis	
DncV-QmA-fw	**AATTCCAGCCTCGTTTCTGGACTGCGGGAAGTTTTCAGTACGATAC**[c]
DncV-QmA-rv	**GTATCGTACTGAAAACTTCCCGCAGTCCAGAAACGAGGCTGGAATT**[c]
DncV-AIA-fw	**CTTTTCATCCGGGGCAGGAAATGGCAATTGCGGATGGCACCTACATG CCC**[c]
DncV-AIA-rv	**GGGCATGTAGGTGCCATCCGCAATTGCCATTTCCTGCCCCGGATGAA AAG**[c]
*Gene expression by q***RT-PCR**	
fliC-qRT-fw	**ATCAAATCCCGTCTCGATG**
fliC-qRT-rv	**GTTTGACCAT CATTCGCAC**
fliA-qRT-fw	**AATAACAGCCAGCTCTTCTC**
fliA-qRT-rv	**GATTACTGTC CAGCAGTTGT**
flhD-qRT-fw	**GTCCGCTATGTTTCGTCTC**
flhD-qRT-rv	**GGTTTCTGCC AGCTTAACC**
CsgD-qRT-fw	**ATCGCTCGTTCGTTGTTC**
CsgD-qRT-rv	**CTGAGGTTAT CGTTTGCCC**
rpsV-qRT-fw	**CAGGCACGTCATATTCTTG**
rpsV-qRT-rv	**GTTGGGTTATTTACCACGC**
Confirmatory sequencing	
pBAD30-fw	**GTCTATAATCACGGCAGAAAAGTCCAC**
pBAD30-rv	**CTGTTTTATCAGACCGCTTCTGC**
DncV-seq	**TACATGCCCATGACGGTGTT**

Underlined indicates [a]restriction sites, [b]regions complementary to pSRKGm, [c]mutated codons.
